# Harnessing Bacterial Lipid Coatings on Gold Nanoparticles for Enhanced Cell Adhesion Applications

**DOI:** 10.1002/smsc.202500584

**Published:** 2026-02-17

**Authors:** Soroosh Gharehgozlo, Shakil Ahmed Polash, Kalpani A. Mirihana, Jakub Matusiak, Lauren Giles, Pierre Vaillant, Shreehari Kodakkat, Serena Ch'ng, Rowan Penman, Samuel Cheeseman, Lydon Alexandrou, Jitraporn Vongsvivut, Andrew J. Clulow, Andrew J. Christofferson, Paul A. Ramsland, Saffron J. Bryant, Aaron Elbourne

**Affiliations:** ^1^ School of Science STEM College RMIT University Melbourne VIC 3001 Australia; ^2^ Department of Construction Materials Engineering and Geoengineering Faculty of Civil Engineering and Architecture Lublin University of Technology Nadbystrzycka 40 20‐618 Lublin Poland; ^3^ Department of Biomedical Engineering Faculty of Engineering and Information Technology The University of Melbourne Parkville VIC 3010 Australia; ^4^ The Graeme Clark Institute The University of Melbourne Parkville VIC 3010 Australia; ^5^ Australian Synchrotron ANSTO 800 Blackburn Road Clayton VIC 3168 Australia; ^6^ Department of Immunology Monash University Melbourne VIC 3004 Australia; ^7^ Department of Surgery Austin Health University of Melbourne Heidelberg VIC 3084 Australia

**Keywords:** bacterial membrane, biomimetic coating, cell adhesion, gold nanoparticle, lipid‐coated particles, uptake

## Abstract

Antimicrobial resistance (AMR) is a global health challenge responsible for millions of deaths annually. Hence, there is an urgent need for improved strategies to combat AMR. Nanoparticle (NP)‐based drug delivery has shown promise for enhancing the efficacy of conventional antibiotic treatments. Moreover, lipid functionalization of NP surfaces can enhance drug loading, colloidal stability, and specificity. Cell membrane vesicles as the outer shell coating for NPs provokes a unique interaction between fabricated NPs and their respective parent bacteria. Despite numerous studies having investigated the use of bacterial extracellular vesicle coatings for drug delivery, to the best of our knowledge, the potential of isolated bacterial membrane lipids has not yet been explored. This study investigates how particle‐cell adhesion changes when gold NPs (AuNPs) coated with bacterial membrane lipids are re‐introduced to their original parent cells. Hence, bacterial lipid coated AuNPs (BLC‐AuNPs) were constructed using AuNPs in conjunction with membrane lipids harvested from *Escherichia coli*. Compared with bare AuNPs, BLC‐AuNPs showed significant increase in particle‐cell adhesion upon re‐exposure confirmed by confocal and electron microscopy. Lipid coating also improved particle distribution and surface coverage on bacterial cells. These findings suggest that bacterial membrane lipid coating provides an effective biomimetic strategy, enhancing drug deliveryto drug resistant pathogens.

## Graphical abstract

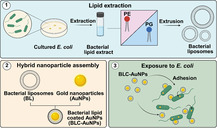

## 1 Introduction

The global misuse and overuse of antibiotic drugs has led to the emergence of antimicrobial resistance (AMR) among a variety of pathogenic bacteria, resulting in catastrophic health consequences for patients suffering from infection. The primary cause of this issue is the decline in antibiotic drug efficacy.^[^ ^1^ ^]^ Annually, AMR directly contributes to over 1.27 million deaths globally, according to the World Health Organization (WHO).^[^ ^2^ ^]^ Additionally, a continuation of the current trends of drug resistance in bacteria may heavily affect international trade and the labor market in the long term, prompting economic repercussions which exceed those of a global financial recession.^[^ ^3^, ^4^ ^]^ The WHO has deemed AMR an urgent issue to be dealt with through the implementation of strict action plans and global coordination.^[^ ^2^, ^5^, ^6^ ^]^ To eradicate or reduce the burden of AMR, much of current research has been directed toward the use of nanosized materials as a safe and effective approach to enhance standard antibiotic treatment.

Often, the development of AMR in bacteria is a complex process that varies across different bacterial cultures. Some notable examples include mutations in efflux pump expression, alterations of bacterial target sites or enzymes, or even biofilm formation, limiting the accessibility of antibiotic drugs to the intracellular space.^[^ ^7^, ^8^ ^]^ Nanomaterials offer several unique advantages against drug resistant bacteria. Their large surface‐to‐volume ratio, along with their small size, make them suitable for targeted drug delivery.^[^ ^9^, ^10^ ^]^ Nanoparticles (NPs) can better penetrate the extracellular matrix and facilitate direct contact with the cell wall, bypassing numerous adaptation mechanisms adopted by bacteria.^[^ ^11^ ^]^ In vivo studies utilizing functionalized NPs have so far demonstrated major therapeutic potential against bacterial infection.^[^ ^12^, ^13^, ^14^ ^]^ The exposure of bacteria to inorganic NPs such as silver,^[^ ^15^, ^16^ ^]^ gold,^[^ ^17^ ^]^ zinc oxide,^[^ ^18^ ^]^ and copper^[^ ^19^, ^20^ ^]^ can have antimicrobial effects through the production of reactive oxygen species (ROS) by inducing oxidative stress within microbial cells. Moreover, inorganic NPs can disrupt fundamental cellular processes such as DNA replication and protein synthesis.^[^ ^21^, ^22^ ^]^ Metallic NPs may adhere to the cell wall due to a surface charge difference, causing mechanical disruptions to the cell membrane and resulting in cellular rupture and death.^[^ ^11^ ^]^ NPs can be functionalized with various molecules to improve target specificity, drug efficacy as well as bioavailability during applications of drug delivery.^[^ ^11^ ^]^

Recently, core‐shell hybrid NPs have emerged as a novel approach for drug delivery. Lipid‐coated hybrid NPs combine an inorganic or polymeric core NP with an outer lipid shell to overcome some of the drawbacks associated with either nonhybrid NP system when used in isolation. Benefits of lipid‐coated hybrid NPs include improved drug release, drug loading efficiency, targeting, and phagocytic clearance.^[^ ^23^ ^]^ Phospholipid molecules may self‐assemble or be functionalized onto the surface of the inorganic core NP, providing colloidal stability, as well as supporting drug loading.^[^ ^23^ ^]^ The addition of a lipid‐based outer shell can promote unique particle‐cell interactions during drug delivery to bacteria.^[^ ^23^ ^]^

Outer membrane vesicles (OMVs) are nanosized vesicles secreted naturally from the outer membrane of cells through the process of budding/detaching.^[^ ^24^, ^25^ ^]^ They are predominantly made of the constituents of the bacterial outer membrane containing various proteins, phospholipids and lipopolysaccharides (LPS); however, their exact chemical composition is complex and dependent on various factors such as their cell type, stress conditions, and growth media.^[^ ^26^, ^27^, ^28^, ^29^ ^]^ When utilized for bacterial drug delivery, notably in hybrid NP systems, OMVs, and cell membrane coatings can successfully entrap and deliver antibiotic payloads to cells, resulting in enhanced drug efficacy.^[^ ^24^, ^30^, ^31^, ^32^, ^33^, ^34^ ^]^ A variety of features including both structural and compositional properties of OMVs such as protein, lipid, and enzyme makeup can allow for adherence, uptake or fusion of these vesicles with their parent cells.^[^ ^35^, ^36^, ^37^ ^]^ Although much of current research has emphasized the role of cell envelope components like peptidoglycan, membrane proteins, lipoproteins, and LPS in facilitating interactions between OMVs and their parent cell strain,^[^ ^38^, ^39^, ^40^, ^41^ ^]^ the role of phospholipids within this process remains poorly defined. Given that membrane lipids contribute to various surface properties, hydrophobicity, membrane curvature, and fluidity in OMVs, it is likely that their unique lipid profile contributes to their affinity to parent cells.^[^ ^35^, ^36^, ^42^ ^]^ Hence, by utilizing the unique composition of bacterial membrane lipids as NP coatings, their potential and role in enhancing drug delivery to parent cells can be assessed. To our knowledge, no study has utilized isolated bacterial lipids for drug delivery within a core‐shell hybrid system. If proven effective, isolated bacterial lipids may provide a significantly more cost‐effective, scalable, convenient, and safer approach for antimicrobial drug delivery compared with OMVs.^[^ ^43^, ^44^ ^]^

**Scheme 1:**
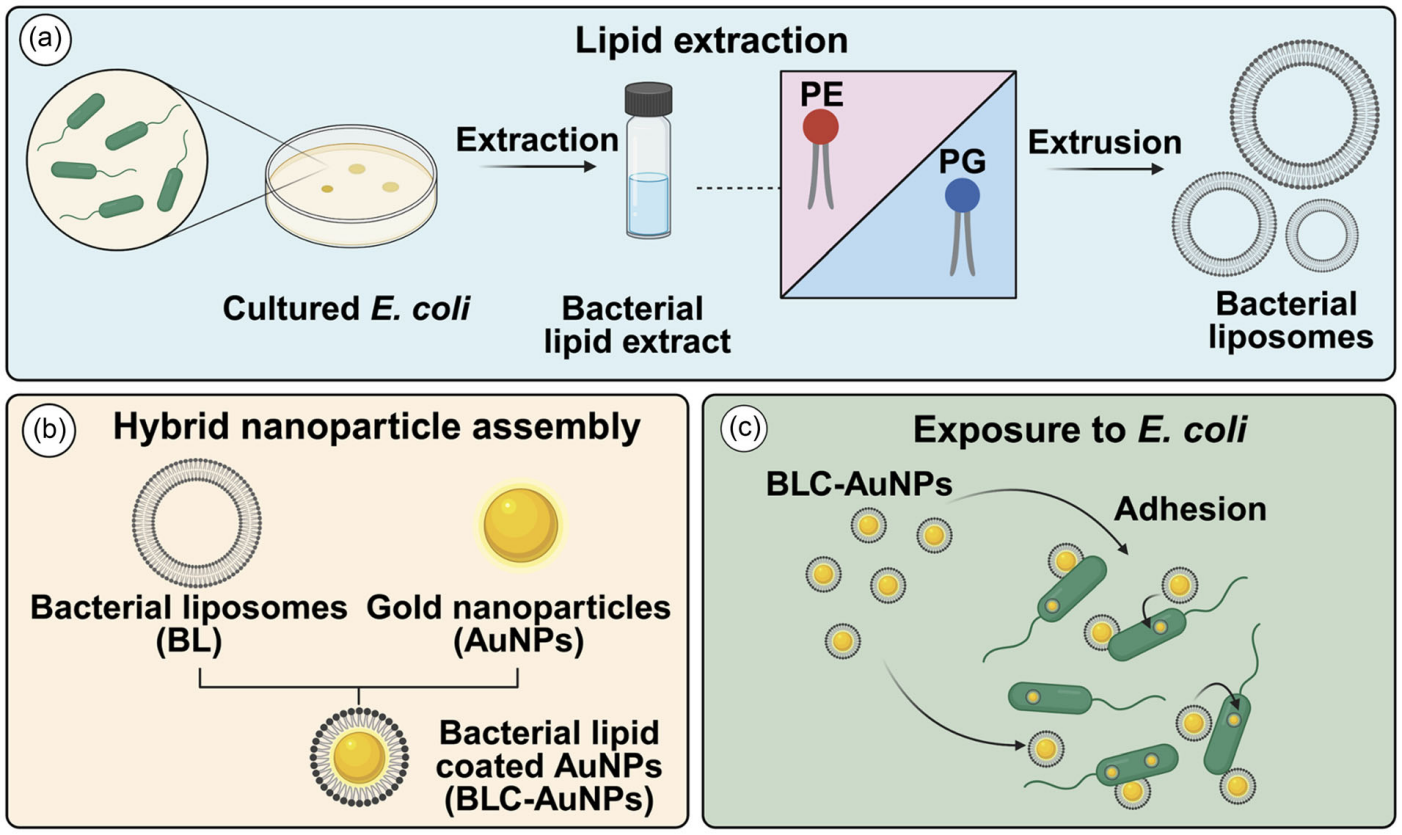
Preparation and application of bacterial lipid–coated gold nanoparticles (BLC‐AuNPs). a) Lipid extraction from *E. coli* bacteria and liposome formation, b) assembly of liposomes with nanoparticles, and c) enhanced adhesion between BLC‐AuNPs and parent cells (*E. coli*) during re‐exposure. Created with BioRender.com.

In this study, membrane lipids were extracted from *Escherichia coli* and used to coat gold NPs (AuNPs), forming a hybrid core‐shell nanodelivery system (BLC‐AuNPs). The BLC‐AuNPs were subsequently re‐introduced to the parent *E. coli* cells and tested for changes in adhesion compared to bare AuNPs (**Scheme** **1**). The use of harvested bacterial lipids as a coating for NPs during drug delivery may facilitate spontaneous particle‐cell adhesion, a process which is rarely observed during conventional bare AuNP drug delivery. This interaction can be modulated by the unique composition of lipids within the *E. coli* membrane, paving the way for the production of simple and convenient biomimetic Trojan‐horse‐like drug delivery systems. The results of this study shed light on the importance of lipid composition during drug delivery and may expand opportunities for exploiting cell membrane‐based lipid NPs to advance AMR therapies.

## 2 Results and Discussion

### 2.1 Extraction and Characterization of Bacterial Lipids

The cell membrane of *E. coli* is composed of a unique array of glycerophospholipids (GP) molecules with various degrees of unsaturation and carbon chain lengths.^[^ ^45^ ^]^ Lipidomic studies of the *E. coli* membrane suggest that phosphatidylethanolamine (PE) and phosphatidylglycerol (PG) are the primary phospholipid components found within the cell membrane.^[^ ^46^ ^]^ Furthermore, the identification of minor lipid classes within the *E. coli* membrane poses a significant challenge as they can be highly dependent on environmental factors, growth conditions, stress adaptation, taxonomic diversity, and metabolic pathways.^[^ ^46^, ^47^ ^]^ Herein, we have used a well‐established lipid extraction technique to isolate the lipids contained within the *E. coli* membrane. The extraction process was designed to isolate membrane lipids and minimize potential impurities. This entailed a slight modification of the Folch method, utilizing a 2:1 v/v chloroform/methanol biphasic system to facilitate the mass transfer of lipids from the aqueous to the organic phase.^[^ ^48^, ^49^ ^]^ In agreement with previously reported findings, mechanical pretreatment using stainless‐steel beads was found to improve miscibility and promote more efficient phase transfer during extraction.^[^ ^50^, ^51^ ^]^ Beads facilitate the disruption of the rigid cell wall and, in turn, improve solvent penetration and overall yield.^[^ ^52^, ^53^ ^]^

**Figure 1:**
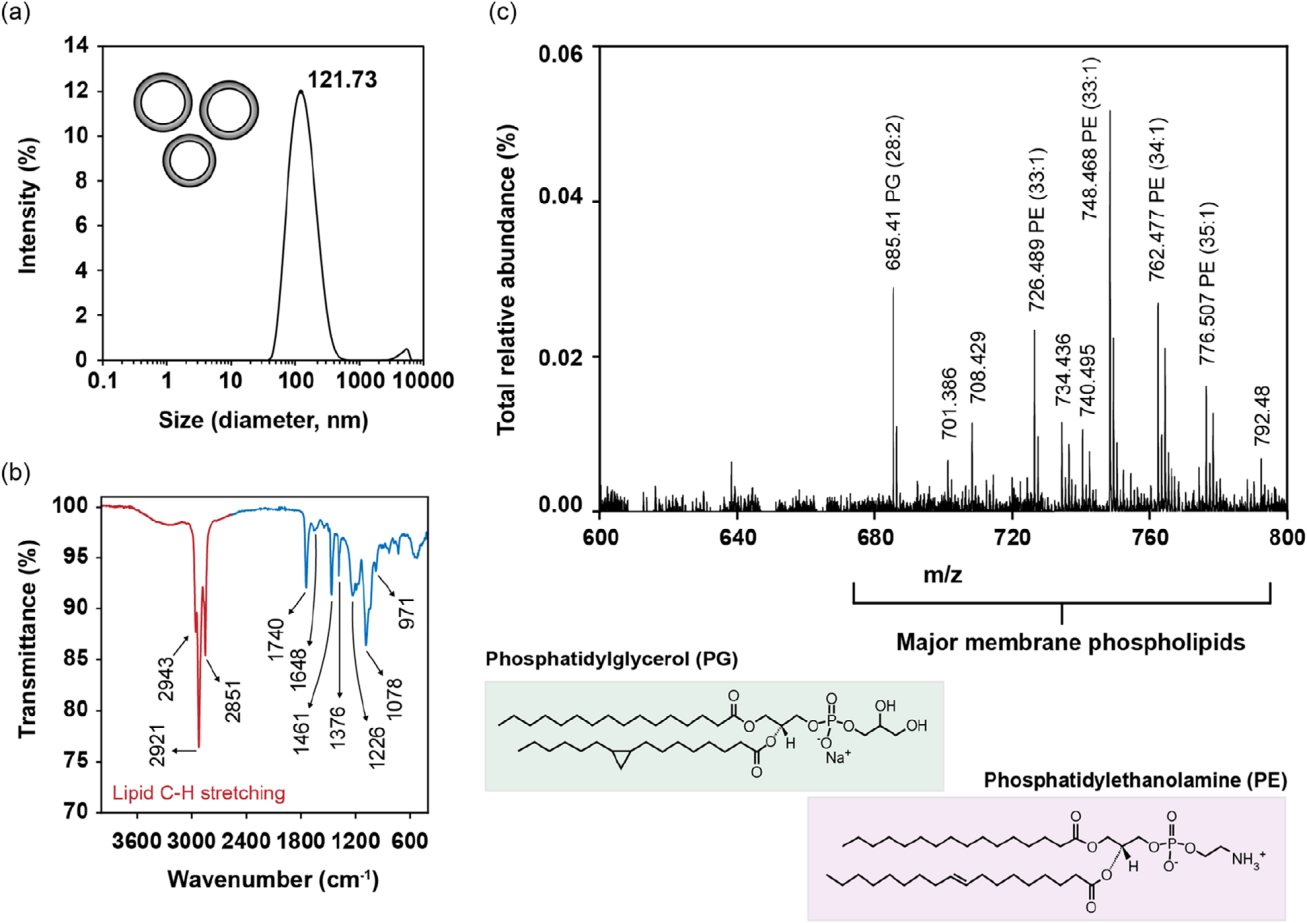
Extraction and characterization of lipids from *E. coli*. a) Hydrodynamic diameter of lipid vesicle using DLS, b) FTIR spectrum of lipid film, highlighting the key absorption bands observed in the bacterial extract, and c) MALDI/TOF chromatograph of extracted lipids, showing identified phospholipids within the 680–780 m/z range. Representative major phospholipids in the *E. coli* membrane are shown as PG (16:0/cy17:0) and PE (16:0/18:1).

Dynamic light scattering (DLS) was used to measure the hydrodynamic diameter of the extruded liposomes in salt buffer solutions. A mean hydrodynamic diameter of 121.7 ± 7.3 nm along with polydispersity index (PDI) of 0.193 ± 0.014 was measured by DLS as shown by the single peak in **Figure** **1**a. The measured lipid vesicle size was well under the indicated pore size (200 nm polycarbonate membrane). Since the measured vesicle diameter is typically expected to fall below the membrane pore size, the discovered values fell within the expected range.^[^ ^54^, ^55^, ^56^ ^]^ Additionally, the mean PDI value indicates that vesicles are sufficiently monodispersed in solution (PDI < 0.2 for low polydispersity).^[^ ^57^, ^58^, ^59^, ^60^ ^]^ Fourier‐transform infrared (FTIR) spectroscopy was employed to identify the characteristic absorption bands of phospholipids in the extracted lipid sample (Figure 1b). Three prominent bands observed at 2943, 2921, and 2851 cm^−1^ correspond to the asymmetric and symmetric stretching modes of ν(C—H) vibrations in the fatty acid tails of phospholipids.^[^ ^61^ ^]^ A strong absorption at 1740 cm^−1^ is attributed to ν(C=O) stretching of ester groups, while a weak band near 1650 cm^−1^ suggests the presence of ν(C=C) bond in the phospholipid structure.^[^ ^62^ ^]^ Additional peaks at 1461 and 1376 cm^−1^ arise from δ(C—H) bending modes of methylene (–CH_2_) groups in acyl chains of lipid bilayers in orthorhombic packing.^[^ ^61^ ^]^ The bands at 1226 and 1078 cm^−1^ are characteristic of phosphate stretching vibrations, confirming the phospholipid backbone structure.^[^ ^63^ ^]^ A distinct peak at 971 cm^−1^ is assigned to ν(C—N) stretching vibration typical of PE.^[^ ^64^ ^]^ It is worth noting that no broad ν(O—H) band was detected in the 3500—2500 cm^−1^ region, indicating the absence of water. This is consistent with successful dehydration of the sample into a thin lipid film. Similarly, the absence of amide bands further confirms that no protein residues were present in the lipid sample extracted from *E. coli*.^[^ ^65^ ^]^

To identify the main phospholipid components within the thin film, mass spectrometry was performed using matrix‐assisted laser desorption/ionization‐time‐of‐flight (MALDI‐TOF) and liquid chromatography–mass spectrometry (LC‐MS). Both PE and PG were identified at roughly similar relative abundance to that of the literature (Figure 1c). These phospholipid peaks are mostly described to reside in the 670–780 m/z region.^[^ ^66^ ^]^ The main [M + H]^+^ peaks include 685 m/z for PG (28:2), 726 PE (33:1), 748 PE (33:1), 762 (34:1), and 776 PE (35:1). These peaks may indicate the presence of PE and PG along with one or two sodium adducts (Na or 2Na + PE/PG). Analysis of membrane lipids in our sample must be further analyzed by other chromatographic procedures to ensure the accuracy of GP identities in the sample. LC‐MS was carried out in negative electrospray ionization (ESI) mode to characterize the presence of phospholipids in the bacterial lipid extract (Figure S1, Supporting Information). Since only ESI was used, little to no fragmentation of the molecular ion was expected within the mass spectrum. Due to the complexity of cell membrane lipids, LC‐MS was only used as a qualitative method to confirm the presence of phospholipid molecules, therefore, internal standards were not prepared for quantitative comparison. The mass spectrum for each peak in the total ion chromatogram was extracted and molecular ions above a nominal cutoff intensity from each spectrum were normalized to their spectrum's base peak (Figure S1, Supporting Information). All base peaks were summed, and molecular ions were represented as a fraction of that grand total. This relative measure does not reflect a quantitative assessment of lipid species; rather, it aims to simplify the dataset to only include the most relevant peaks. The most prominent peaks were found at 702, 716, 688, and 730 m/z corresponding to possible PE lipids and 761 m/z corresponding to the presence of PG lipids (Figure S1, Supporting Information). In general, results favored a larger number of peaks [M‐H]^−^, indicating the possible presence of PE. This may be because of ionization, or solubility in the mobile phase, or methodology during analysis. Further analysis using collision‐induced dissociation (CID) can confirm molecular ion identity through emerging fragmentation patterns.^[^ ^67^ ^]^ However, comprehensive lipidomic study of *E. coli* membrane lipid species is beyond the scope of this study. A strong detection of molecular ions within the 500–800 m/z can suggest the presence of various forms of PE and PG phospholipid species that are commonly found within the *E. coli* membrane.^[^ ^66^ ^]^

The lipid composition plays a significant role in determining the fluidity and ultimately may help explain their adhesion mechanism during particle‐cell experiments.^[^ ^68^ ^]^ The *E. coli* outer membrane largely is composed of PE lipids (≈75%) that are zwitterionic, along with anionic PE (≈20%) and cardiolipin (≈5%), giving cells an overall negative charge.^[^ ^69^, ^70^ ^]^ The combination of various lipid species in the *E. coli* outer membrane can play a crucial role in the membrane structure, surface charge regulation and in fluidity. PE is thought to decrease net negative surface charge in bacteria such as *B. subtilis* and in some instances has been described as a “fusogenic phospholipid,” showing a correlation between high percentage of membrane PE and successful liposome‐bacterial fusion.^[^ ^69^, ^71^ ^]^ While purified phospholipids are commercially available, they are very expensive. In contrast, our bench‐top solvent extraction of bacterial membranes yields a heterogeneous mixture of native phospholipids at a fraction of this cost, without requiring specialized infrastructure. This low‐cost, scalable approach could therefore provide a practical alternative for large‐scale applications, even if individual purified phospholipids exhibit similar physicochemical behavior.

### 2.2 Synthesis and Characterization of Citrate‐Capped AuNPs

**Figure 2:**
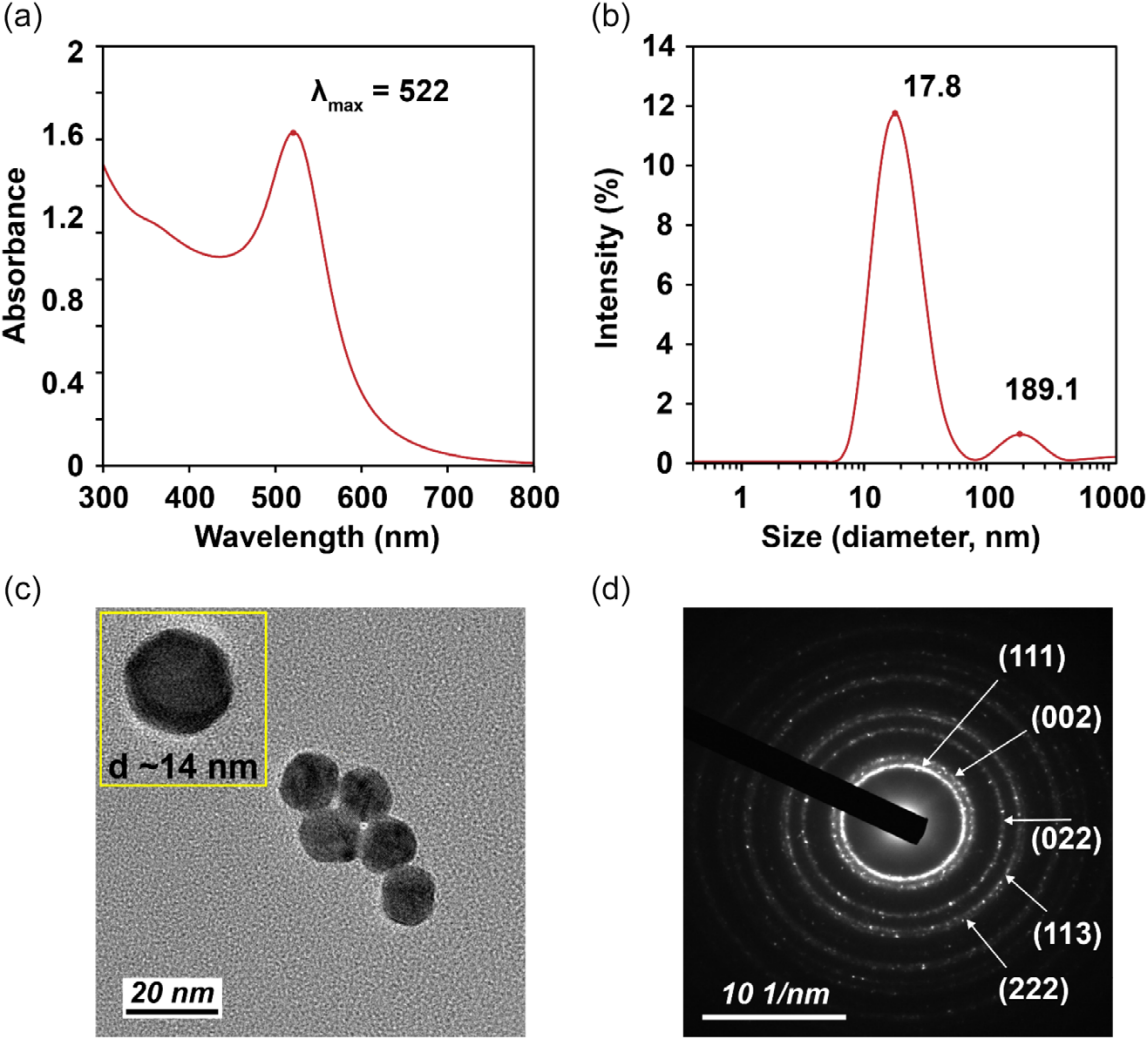
Characterization of citrate‐capped AuNPs. a) UV‐Vis spectroscopy, b) dynamic light scattering, c) TEM, and d) diffraction ring pattern of the synthesized AuNPs.

The core compartment of our core‐shell system is the construction of AuNPs. The core will essentially provide a platform for lipid molecules to deposit onto a solid surface through self‐assembly, establishing a more robust drug delivery system that can be both optically and chemically tracked during particle‐cell exposure. AuNPs were synthesized through a temperature and time‐controlled approach, closely following the Turkevich method.^[^ ^72^, ^73^ ^]^ To ensure the size of the particles was small (<20 nm) and monodisperse, citrate was added at a higher molar ratio (7.87 molar ratio citrate to gold).^[^ ^74^ ^]^ Additionally, the anionic nature of citrate not only reduces particle aggregation but also helps achieve a monodisperse population through particle–particle repulsion to maintain a level of particle stability in solution.^[^ ^75^ ^]^ The suspension of synthesized citrate capped AuNPs was dark red in appearance indicating particle size below 40 nm and stable colloidal dispersion, reflecting minimal aggregation and uniform surface plasmon resonance (SPR).^[^ ^76^ ^]^ As shown in **Figure** **2**a, the ultraviolet‐visible (UV‐Vis) absorption spectrum of the synthesized AuNPs displayed a maximum peak absorbance of 522 nm corresponding to spherical AuNPs ranging from 10–40 nm in diameter.^[^ ^77^ ^]^ The position and intensity of the SPR peak are highly sensitive to particle size, shape, and the surrounding dielectric environment. Generally, a red shift (i.e., a shift of the peak toward longer wavelengths) in SPR bands >540 nm is associated with an increase in particle size. This effect is attributed to the reduction in collective electron oscillation frequency leading to a lag in electromagnetic response and a larger SPR absorption wavelength during characterization.^[^ ^77^ ^]^

Next, DLS was used to measure the hydrodynamic diameter of the synthesized AuNPs. A mean hydrodynamic diameter of 20.8 ± 2.4 nm, along with a peak intensity reading of 17.8 nm was read for AuNPs from DLS measurements (Figure 2b), falling within the range estimated by UV‐Vis spectroscopy. A calculated polydispersity index (PDI) of 0.30 ± 0.04 from DLS suggests slightly polydisperse population of AuNPs in solution (ideal PDI < 0.2). Despite having a narrow size distribution, the second AuNP population in the DLS measurements at ≈190 nm may have contributed to an increased PDI value. This is likely due to AuNP aggregates. To confirm the morphology, size distribution, and crystallinity of the synthesized AuNPs, transmission electron microscopy (TEM) and selected area electron diffraction (SAED) images were taken of the AuNPs (Figure 2c,d). The synthesized AuNPs were found to be spherical in morphology, containing smooth but defined edges (Figure 2c). Additionally, little polydispersity is observed across populations. A count of randomly chosen 364 AuNP sizes led to the distribution graphed in SI (Figure S2, Supporting Information). The mean particle diameter was found to be 13.3 ± 1.7 nm. The difference in particle diameters reported from the DLS and TEM can be attributed to the inherent principles and limitations of each technique. DLS measures the hydrodynamic diameter that encompasses not only the particle core but also the surrounding solution layers, surface ligands, or weakly associated molecules. Additionally, DLS is highly sensitive to larger particles or aggregates, as light scattering intensity scales with the sixth power of particle size. This causes slightly bigger or aggregated particles to become over‐represented in the DLS data due to their increased scattering properties. In contrast, TEM provides a direct visualization of the physical size of dried particles with higher accuracy and was therefore utilized in this study. Finally, the SAED of a selected particle was taken. The diffraction pattern followed a series of defined rings verifying a polycrystalline structure (Figure 2d). A measurement of *d*‐spacing values and their corresponding miller indices (*hkl*) has been summarized (Table S1, Supporting Information). The measured interplanar spacing was in accordance with face‐centered cubic (FCC) gold atoms.

### 2.3 Synthesis and Characterization of Bacterial Lipid Coated‐AuNPs (BLC‐AuNPs)

AuNPs were combined with the appropriate amount of lipids, enough to coat the surface of all NPs with respect to surface area calculations (Table S2, Supporting Information). This approach was also cross referenced with the color of each solution to confirm that a transition of red to blue color, which would indicate severe particle aggregation, did not take place. Vesicle fusion as described by Jiang et al. was accomplished through bath ultrasonication of AuNPs combined with bacterial lipid vesicles.^[^ ^23^ ^]^ Maintenance of temperatures higher than the transition temperature (T_m_) of the bacterial lipids was thought to help facilitate lipid adsorption to AuNP and assist lipid‐particle self‐assembly during ultrasonication.^[^ ^78^ ^]^

**Figure 3:**
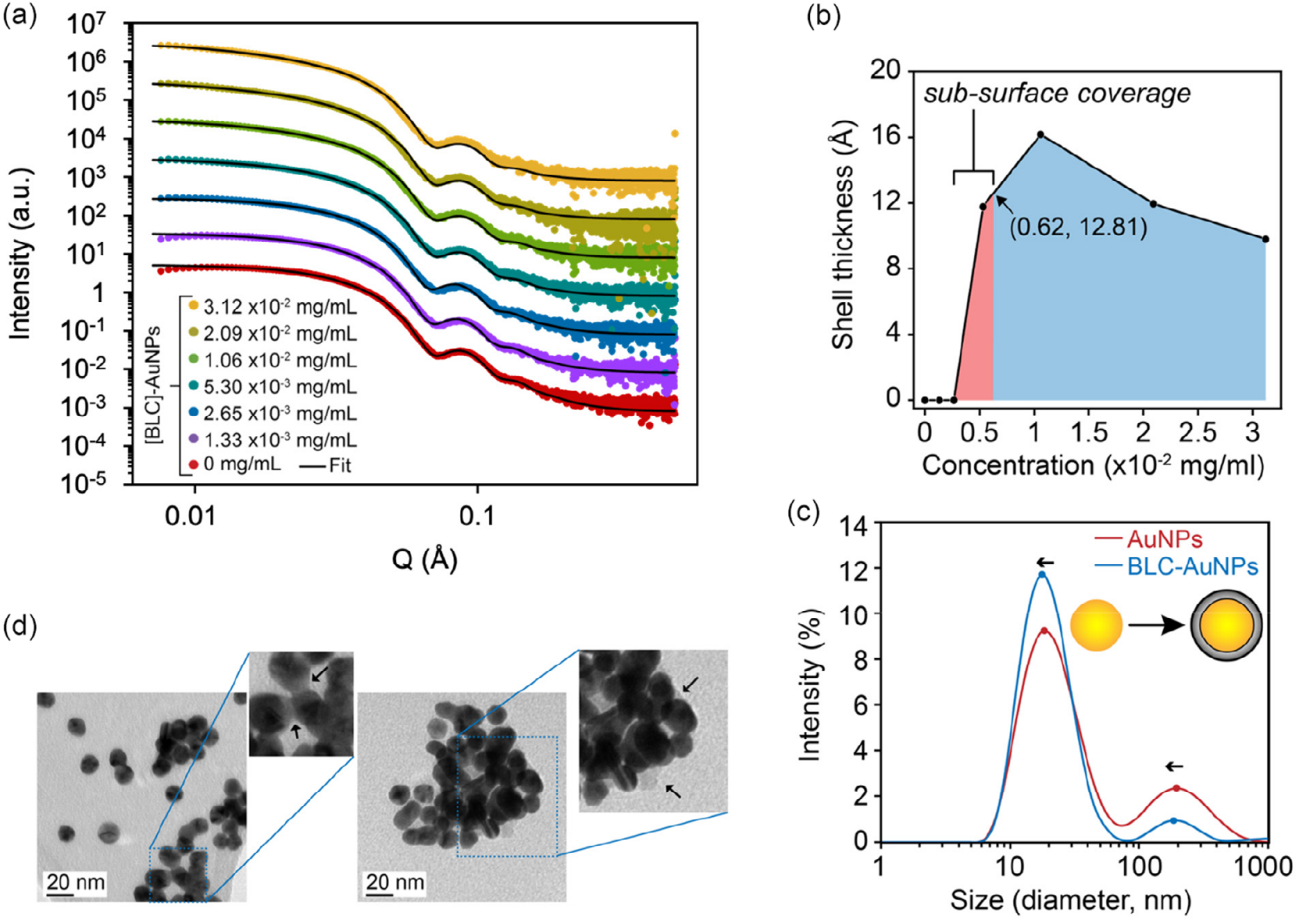
Characterization of BLC‐AuNPs and the formation of a lipid shell. a) Synchrotron SAXS data of AuNPs and BLC‐AuNPs with different concentrations of bacterial lipids (1.33 × 10^−3^ to 3.12 × 10^−2^ mg mL^−1^) with overlaid best fit obtained using SasView's core‐shell fractal model. For clarity, data at different concentrations are offset by a factor of ×10^*n*^ (where *n* = 1–7 for 1.33 × 10^−3^ to 3.12 × 10^−2^ mg mL^−1^ BLC‐AuNPs respectively). b) Shell thickness obtained from the core‐shell fractal model. The vertical red line indicates the concentration of lipids required to obtain full surface coverage of AuNPs in solutions. c) DLS of bare AuNPs and BLC‐AuNPs, highlighting a shift in mean particle diameter. d) TEM of BLC‐AuNPs. The arrows indicate the lipid layer around the AuNP surface.

The configuration of lipid molecules on the AuNPs surface, as well as the potential formation of a lipid corona surrounding the AuNPs, was investigated by small‐angle X‐ray scattering (SAXS) at the BioSAXS beamline at the Australian Synchrotron (ANSTO). Fitting the SAXS profiles of the BLC‐AuNPs provided information on the formation of a lipid shell on the AuNP surfaces with increasing concentrations of bacterial lipids in solution (**Figure** **3**a). To observe changes in particle‐lipid configuration on the AuNP surfaces, bacterial liposomes were incrementally added to the suspension of AuNPs circulating through the X‐ray beam path. The rapid increase in shell thickness prior to the calculated full coverage concentration suggests that subsurface coverage concentrations form a partial shell that rapidly grows until full coverage is achieved. This process also helped to shed light on particle aggregation, as well as provide a potential estimate of the BLC‐AuNP shell thickness at various concentrations of lipids in solution (Table S3, Supporting Information). The resulting changes in scattered X‐ray intensity as a function of the modulus of the scattering vector (Q) were fitted using the core‐shell fractal and sphere models from SasView. The core‐shell fractal model gave the lowest *χ* ^*2*^ (chi‐squared) error value, as well as the best visual fit when compared with sphere, core‐shell sphere, and regular fractal models in SasView. The fitted core AuNP radius acquired from the sphere model corresponded to a particle diameter of 12.4 nm. This is consistent with the particle size measured by TEM (particle diameter = 13 ± 2 nm).

With sequential additions of lipids to the AuNPs, a gradually increasing upturn of the low Q region of the scattering profiles was observed. This upturn indicates a level of particle aggregation occurring with increasing concentrations of bacterial lipids in the AuNP solution. To choose the most appropriate model, both spherical and core‐shell spherical models were attempted, as well as the default fractal model with only limited success at reproducing consistent fits across the various samples. The spherical model was capable of fitting particles with lipid concentrations at or under 2.65 × 10^−3^ mg mL^−1^ but failed to capture the effects of particle aggregation in the low Q region of the profiles with increasing lipid concentrations. Moreover, higher Q features associated with surface details were also not captured by the basic sphere model. In contrast, the default and core‐shell fractal models (modelling a fractal aggregate constructed from base spheres) were able to account for aggregation effects observed in the low Q region of the scattering profiles. When the volume fraction of particles in the system (*ϕ*) was unconstrained, both fractal and sphere models demonstrated a decreasing volume fraction as a function of lipid concentration. This decrease in volume fraction was not reflected in the SPR of the prepared particles, as the lipid concentrations used for all particle‐cell experiments yielded a negligible change in UV‐vis spectra compared to bare AuNPs (Figure S4, Supporting Information). Additionally, the BLC‐AuNP solutions retained a dark red color upon lipid addition and sonication and heating at concentrations used for particle‐cell experiments. However, some particle settling was observed which can explain the decrease in particle volume fraction observed within the model fits (Figure S3, Supporting Information). The *χ* ^2^ values for all fits indicated that both statistically and visually, the core‐shell fractal model provided the best fit with the lowest error, while the basic sphere model better described AuNP samples containing sub‐surface coverage lipid concentrations. Hence, the core‐shell fractal and sphere models were chosen to represent the best fit for our data accordingly (Figure 3a).

The core‐shell fractal model in SasView comprises a core‐shell sphere from factor *P(q)* and the Teixeira fractal structure form *S(q)* as follows.

(1) $$P\left(q\right)=\frac{\phi }{V_{s}}[3V_{c}\left(\rho_{c}-\rho_{s}\right)\frac{\sin\left(qr_{c}\right)-qr_{c}\cos\left(qr_{c}\right)}{{\left(qr_{c}\right)}^{3}}+{3V_{s}\left(\rho_{s}-\rho_{\text{solv}}\right)\frac{\sin\left(qr_{s}\right)-qr_{s}\cos\left(qr_{s}\right)}{{\left(qr_{s}\right)}^{3}}]}^{2}$$

(2) $$S\left(q\right)=1+\frac{D_{f}\Gamma \left(D_{f}-1\right)}{{\left[1+1/{\left(q\xi \right)}^{2}\right]}^{\left(D_{f}-1\right)/2}}\frac{sin\left[\left(D_{f}-1\right)\tan^{-1}\left(q\xi \right)\right]}{{\left(qr_{s}\right)}^{D_{f}}}$$

here, volume fraction is *ϕ*, fractal dimension represented by *D*_f_, and the correlation length is given by *ξ*. Unlike the default fractal model, the core‐shell fractal model also includes a thickness parameter, with a corresponding shell scattering length density (SLD), as well as core and solvent SLDs (given by *ρ*_c_, *ρ*_s_, and *ρ*_solv_), enhancing the accuracy of the fit for hybrid core‐shell particles.^[^ ^79^ ^]^ A basic spherical model was able to describe AuNPs well and yielded a radius of 62.1 Å. This radius was used as an initial parameter for our core‐shell fractal fitting.^[^ ^80^, ^81^ ^]^

The following default parameters were used for the core‐shell fractal models: radius = 62.1 Å, shell SLD = 7.73 × 10‐6 Å^2^, fractal dimension *D*_f_ = 1.5, volume fraction *ϕ* = 4 × 10^−4^, and correlation length *ξ* = 100 Å. The default shell‐SLD value was determined using the SasView SLD‐calculator, where the density of lipids was estimated using the mass and volume of POPE surrounding each AuNP (area per molecule = 0.72 nm^2^).^[^ ^82^ ^]^ Fractional dimension was estimated at 1.5 since our system contains 3D spherical particles and therefore must be between 1 and 3 in fractional dimension value.^[^ ^79^ ^]^ Correlation length (*ξ*), volume fraction (*ϕ*) and shell thickness were left unconstrained, with shell‐SLD to vary between values of 6.5–8 Å^2^ during fitting to capture any variation in parameters across our samples. The radius, SLD‐core (*ρ*c), SLD‐solvent (*ρ*solv), and Df were fixed during fitting, as these parameters were not projected to change significantly across samples.

The resulting fits show a sharp increase in shell thickness from bare AuNPs to 5.30 × 10^−3^ mg mL^−1^ BLC‐AuNPs, with the projected shell thickness reaching a maximum of 16.2 Å at 1.06 × 10^−2^ mg mL^−1^ and slightly declining to 9.8 Å at the highest lipid concentration (3.2 × 10^−2^ mg mL^−1^) (Figure 3b). According to our NP surface calculations, it is estimated that the total concentration of lipids required to achieve full coverage of the AuNP particles in solution was 0.62 × 10^−2^ mg mL^−1^, as described by the vertical line in (Figure 3b). The corresponding shell thickness at full coverage is therefore estimated at 12.8 Å. Since the thickness is found to grow up to lipid concentrations of 1.06 × 10^−2^ mg mL^−1^, our calculated concentration for total lipid coverage may have been underestimated. However, since a (5.2 ± 1.5)‐fold excess of lipids were actually used in particle‐cell experiments, the sufficient coverage of all AuNPs by bacterial lipids was ensured. Studies using neutron reflectometry (NR) and X‐ray reflectometry suggest that a POPE monolayer under constant 20 mN m^−1^ pressure contains an acyl chain and headgroup layer thickness of 13.6 and 6.8 Å respectively. Therefore, within these ranges, the nature of our BLC‐AuNP shell coating closely favors a monolayer rather than a bilayer.^[^ ^83^ ^]^ Considering that the lipid shell on our BLC‐AuNPs contain a variety of lipid lengths and species (e.g., PE, PG and CL), the orientation of those lipid molecules on the AuNP surface (vertical or tilted) can influence the measured shell thickness. This can explain the slight variation observed between our SAXS‐measured thickness and the thickness of pure phospholipids such as POPE. The results obtained are in agreement with literature, since without the support of a robust inner layer on the surface of AuNPs such as thiol linkers, the spontaneous formation of a bilayer on 13 nm AuNPs, although not impossible, can be energetically unfavorable.^[^ ^84^, ^85^ ^]^

The core‐shell fractal model suggests that the shell thickness continuously increases upon the addition of bacterial lipids; however, the projected thickness may decline after all particles are sufficiently coated. The growth pattern of shell thickness under the concentrations needed for full AuNP coverage signals the rapid formation of the suspected lipid monolayer. Additionally, above full coverage concentrations, the stagnation and decline of shell thickness may be described by a state of equilibrium being reached between the AuNPs and lipids; however, more research beyond the scope of this study is required to better understand the particle‐lipid behavior at these concentrations. MD simulations in a later section predict a tilted orientation for the majority of lipids on AuNPs when fully covered; however, the exact configuration of lipids at excess concentrations will require further comprehensive MD simulations beyond the scope of the current study. Interestingly, the basic sphere model showed a lower *χ* ^2^ error compared with core‐shell fractal and core‐shell sphere models for BLC‐AuNPs containing lipid concentrations at or under 2.65 × 10^−3^ mg mL^−1^. However, when using the sphere model, the differences between error values grew in samples above lipid concentration of 2.65 × 10^−3^ mg mL^−1^, with the core‐shell fractal model offering the best overall fit at these concentrations. This can indicate that at concentrations at or under 2.65 × 10^−3^ mg mL^−1^, more basic core‐shell sphere models better describe interparticle behavior due to low levels of particle aggregation. Above 2.65 × 10^−3^ mg mL^−1^, the deviation between measured and fitted curves becomes more apparent in the core‐shell sphere fits, while core‐shell fractal fits showed consistent and stable SLD‐shell estimations and a lower chi‐squared error (Table S4 and S5, Supporting Information). Correlation‐length values (*ξ*), representing the upper limit for cluster/aggregate sizes, fitted below the radius of core particles in samples below lipid concentrations of 2.65 × 10^−3^ mg mL^−1^. Since the fractal core‐shell model combines the core‐shell spherical form factor *P(q)* with the Teixeria fractal structure factor *S(Q)*, the value of *ξ* < core radius can indicate that the fit is more *P(q)* dominant and may show negligible fractal behavior. Otherwise, fractal behavior may be present at smaller cluster sizes in solution.

The hydrodynamic diameter of the BLC‐AuNPs was measured at the concentrations used in particle‐cell experiments (2.909 × 10^−2^ mg mL^−1^) using DLS (Figure 3c). Due to the high variability of lipid content across batches, a (5.2 ± 1.5)‐fold excess of the estimated minimum concentration of lipids for full AuNP surface coverage was used to ensure the adequate coating of particles without excessive aggregate formation. A mean hydrodynamic diameter of 22.9 ± 1.8 nm was measured for the standard lipid concentration BLC‐AuNPs used for all particle‐cell experiments. The hydrodynamic diameter of BLC‐AuNPs was 2.4 ± 1.2 nm larger than bare AuNPs as observed by DLS. This increase in diameter is consistent with the shell thickness values estimated from SAXS at full lipid coverage concentrations. Therefore, both DLS and SAXS data are in agreement on the formation of a lipid monolayer on BLC‐AuNPs. PDI was measured as 0.4 ± 0.02 for BLC‐AuNPs, indicating a slightly increased polydispersity compared to bare AuNPs due to aggregate formation. Two primary populations were identified in all DLS measurements on AuNPs and BLC‐AuNPs. The lower diameter peak (18.7 nm) is thought to reflect a single population of coated BLC‐AuNPs, whereas the peak at larger particle size is weaker in intensity and likely results from the aggregation of particles in solution observed by SAXS. A mild elevation of the baseline for BLC‐AuNPs near 1000 nm indicates the presence of some larger particles or aggregates in the solution. BLC‐AuNP samples demonstrated a positive shift in mean hydrodynamic diameter when compared to bare AuNPs, potentially indicating the formation of lipid coronas around the surface of AuNPs. However, since DLS tends to have a bias for large particles, the exact measurement of the lipid coating would require further microscopic analysis. TEM results suggest that particles remain intact after lipid coating and layer of lipid is visible around the AuNP surface (Figure 3d). The extent of particle aggregation was further investigated through UV spectroscopy (Figure S4, Supporting Information). No changes in the UV spectrum of BLC‐AuNPs were observed at the lipid concentration used for experiments. The color of the BLC‐AuNP solution at these concentrations was deep red, indicating the presence of stable particles and minimal aggregation and precipitation at lipid concentrations 5.2 ± 1.5 times the amount needed for full particle coverage (Table S2 & S6, Supporting Information). Additionally, no significant change in zeta potential was noticed in the AuNPs after lipid coating (Figure S5, Supporting Information).

### 2.4 Exposure of BLC‐AuNPs to Bacteria

After the incubation of *E. coli* cells with both bare AuNPs and BLC‐AuNPs, the interaction between particles and cells was visualized and characterized using both physical and chemical methods such as Confocal laser scanning microscopy (CLSM), high‐resolution synchrotron‐based ATR‐FTIR, and scanning electron microscopy (SEM). This study primarily aims to assess the impact of bacterial membrane lipid coatings on NP drug delivery. Therefore, uncoated AuNPs serve as the most optimal baseline control, allowing us to observe the relative enhancements in particle‐cell adhesions compared with BLC‐AuNPs. Successful particle‐cell adhesion can set the stage for future investigations into how the lipid composition of both bacterial membranes and model lipids affect the mechanism and extent of drug delivery.

**Figure 4:**
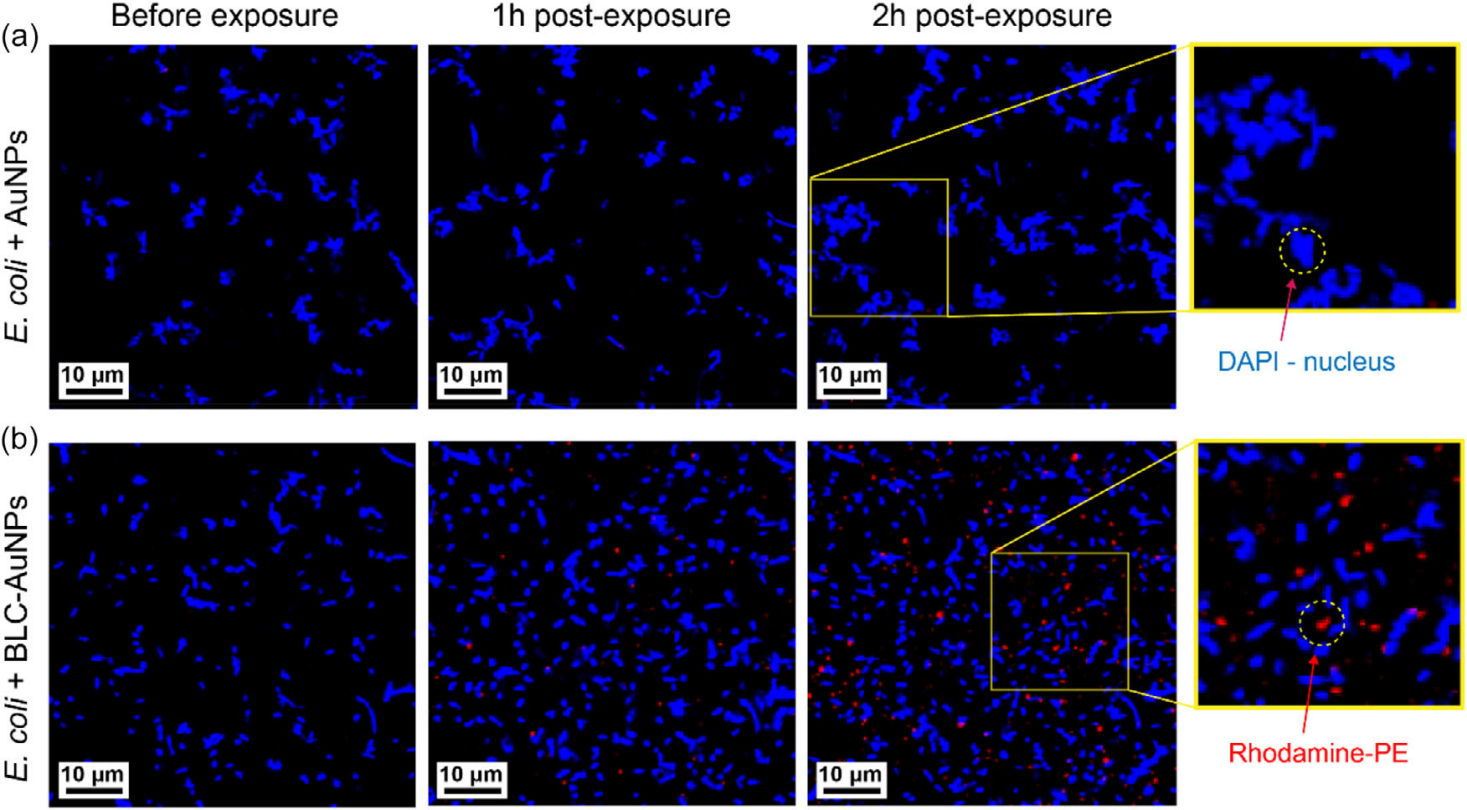
CLSM images of *E. coli* cells (blue, DAPI) prior to exposure of a) AuNPs and b) BLC‐AuNPs (red, Rhodamine‐PE) along with images at 1 and 2 h post particle‐cell exposure.

During the preparation of BLC‐AuNPs, Rhodamine‐PE (Rh‐PE) was embedded within the bacterial vesicles prior to the AuNP‐liposome vesicle fusion. This step ensured the fluorescence of the BLC‐AuNP shell during CLSM experiments.^[^ ^86^ ^]^ Since AuNPs can auto‐fluoresce red, large accumulations of AuNPs should have been captured throughout the duration of the scan, and their absence can suggest minimal to no adhesion occurring between bare AuNPs and cells (**Figure** **4**a).^[^ ^87^, ^88^ ^]^ Therefore, intensity of red‐light emission from the Rh‐PE was compared before and after exposure at 1 and 2 h intervals at 620 nm (Figure 4a,b). Prior to particle exposure, little to no red‐light intensity other than background noise was identified in all samples. Every attempt was made to ensure the scanning area remained stable during particle addition, which can help track the coating pattern of particles onto the cells. Within 1 h after particle exposure, no changes could be seen in the control group; however, BLC‐AuNPs show signs of red fluorescence surrounding *E. coli* cells (Figure 4). The intensity of the red fluorescing Rh‐PE was found to increase as a function of time. At 2 h post‐exposure, the BLC‐AuNPs sample showed intense fluorescence surrounding many cells (Figure 4b). Within proximity to *E. coli* cells, spherical patterns started to emerge and grow in intensity as seen in Figure 4b. These patterns likely indicate the accumulation of BLC‐AuNPs, or Rh‐PE containing lipid vesicles. The same pattern of particle deposition was not found in the control sample cells exposed to bare AuNPs. Despite the presence of red fluorescence around many cells, the pattern of fluorescence appeared to be non‐uniform, possibly indicating the clustering of BLC‐AuNPs. No such pattern was observed in the control group prior and after bare AuNPs exposure. It is important to note that the CLSM instrument has a lateral detection limit of 140 nm, which is greater than the expected BLC‐AuNP particle size and thus may be limited in its capability to detect sub‐20 nm BLC‐AuNPs. Therefore, CLSM may be prone to highlighting larger BLC‐AuNP aggregates or clusters, rather than individual NPs. Thus, our findings indicate that AuNPs coated with isolated *E. coli* membrane lipids (BLC‐AuNPs) more readily adhere to *E. coli* during re‐exposure, compared with uncoated AuNPs.

### 2.5 Particle‐Cell Interaction

**Figure 5:**
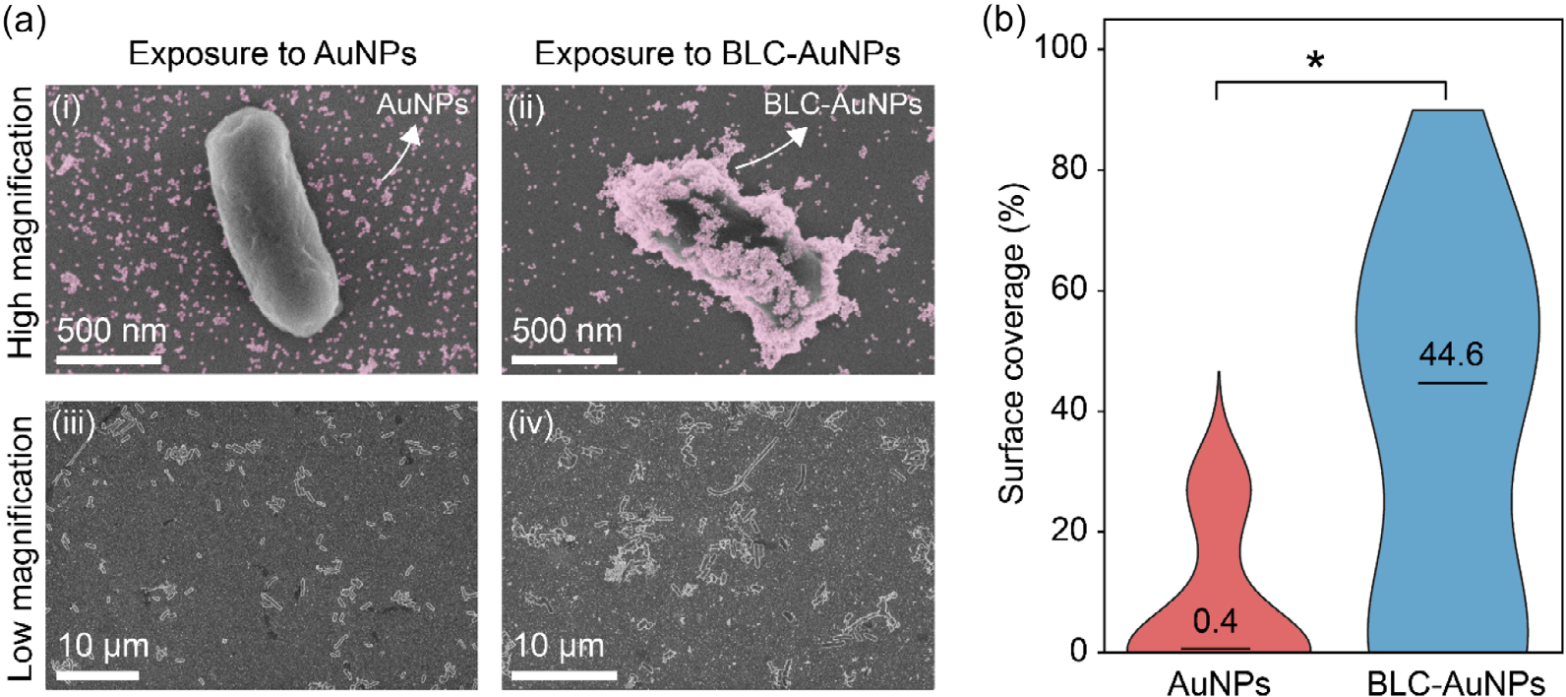
Particle‐cell interaction studied using SEM at 2 hr post‐exposure. a) Highlights differences in coating pattern between *E. coli* cells exposed to Bare AuNPs (I and III) and BLC‐AuNPs (II and IV). b) Median of cell surface coverage (%) (at 2 h) between populations of bare AuNPs and BLC‐AuNPs demonstrating a significant (*p* = 0.0325) increase in bacterial surface coverage when cells were exposed to BLC‐AuNPs.

In CLSM, the red and blue channels suggested BLC‐AuNPs deposition near the cells. However, the limited signal overlap highlighted the need for SEM imaging, as CLSM may lack the resolution to detect sub‐20 nm particles on the cell surface. Therefore, a similar methodology to CLSM was carried out under SEM to visually inspect the interaction between BLC‐AuNPs and *E. coli* in solution after 2 h of exposure. SEM results found a significant variation in adhesion pattern and surface coverage between cells exposed to BLC‐AuNPs and bare AuNPs. Control *E. coli* cells had a smooth appearance with little damage observed to the cell population. During exposure to AuNPs, cells were mostly unchanged in terms of appearance; however, aggregation and lysis were more prevalently observed, as shown in the overview (**Figure** **5**aiii). The presence of 10–15 nm AuNPs is known to induce mechanical stress on the cellular membrane and cause a degree of damage to nearby cells.^[^ ^89^, ^90^ ^]^ Cell damage leads to the release of intracellular contents and thus results in a cascade accumulation of AuNPs. However, the extent of this accumulation looks to be minimal from the SEM images obtained (Figure 5a). The pattern of coating in cell samples treated with bare AuNPs resembles random focal accumulation of nanoparticles and was rarely found to adhere to the top half of the cells. Most AuNPs resided on the background silicon or had accumulated near lysed cells or surrounding the cell‐silicon contact area (Figure 5a(i and iii)). The surface area occupied by BLC‐AuNPs and bare AuNPs was expressed as a percentage of the entire cell surface area and compared between the two samples. The surface coverage percentage of the two sample populations (*n*_BLC‐AuNPs_ = 13, *n*_AuNPs_ = 13) was compared using the Mann‐Whitney U‐Test (*U‐value = *44, *Z‐value = *−2.138, *p = *0.0325) (Figure 5b). Compared with bare AuNPs exposed to cells, those exposed to BLC‐AuNPs showed a significant increase in particle surface coverage. This significant increase can highlight the impact of bacterial lipid addition during particle‐cell interaction. On the other hand, cells exposed to BLC‐AuNP samples displayed a distinct pattern of coating in contrast to bare AuNPs. BLC‐AuNPs tended to cover the cell surface, particularly in regions not exposed to the silicon substrate (Figure 5a(ii)). Additionally, BLC‐AuNP treated cells displayed a higher level of cell clumping on overview scans when compared with both untreated control (Figure S6, Supporting Information) and bare AuNP‐treated samples (Figure 5a(iii–iv)). The consistent pattern of particle coating on cells in BLC‐AuNP‐treated samples may suggest preferential adhesion of bacterial lipid–coated particles to their parent cells. The adhesion of bare AuNPs resembled random particle deposition, rather than target specific adhesion. An approximate 44.2 percentage point increase in median surface coverage (%) with BLC‐AuNPs further confirms that the addition of bacterial lipid coating enhances particle‐cell adhesion and may ultimately boost bacterial cell targeting (Figure 5b and Table S7–S9, Supporting Information).

### 2.6 Synchrotron Macro ATR‐FTIR Spectroscopy

In this study, the *E. coli* cells were incubated for 2 h with either bare AuNPs or BLC‐AuNPs prior to analysis under conditions consistent with the SEM and CLSM experiments. Synchrotron macro ATR‐FTIR microspectroscopy at the infrared microspectroscopy (IRM) beamline of the Australian Synchrotron was then employed to obtain high‐resolution chemical maps of the membrane interface following nanoparticle‐cell exposure. This macro‐ATR‐FTIR technique provides molecular insight into chemical alterations within the bacterial cell membrane induced by nanoparticle treatment.^[^ ^87^, ^91^ ^]^

**Figure 6:**
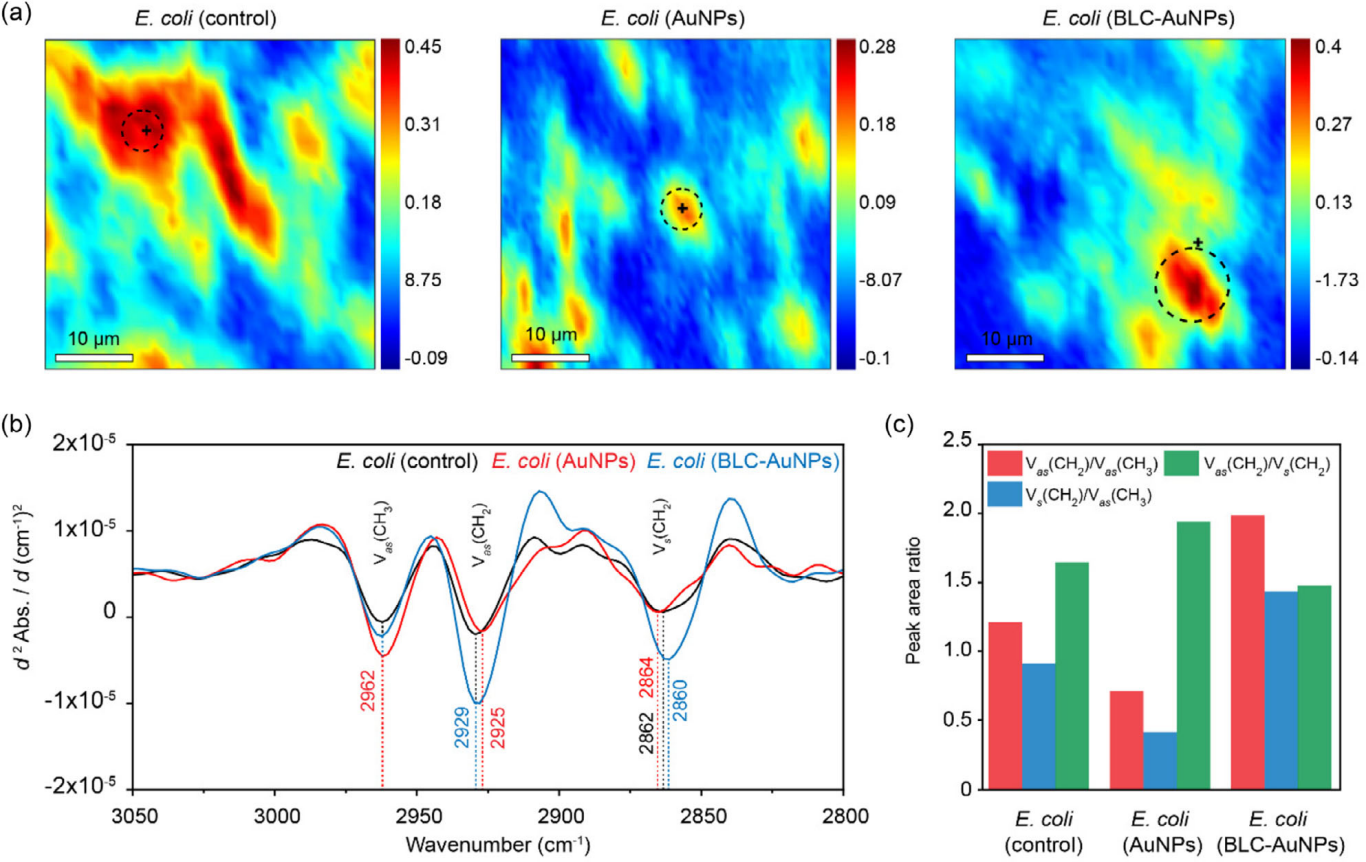
Synchrotron macro ATR‐FTIR chemical maps of untreated and treated E. coli cells. a) Chemical maps of amide I protein distribution observed in control, bare AuNP‐treated, and BLC‐AuNP‐treated cells, with circles indicating the location of the targeted cell. b) Comparison of average inverted second derivative spectra obtained from hierarchical cluster analysis showing spectral variation of ν(C—H) vibrational modes of lipids at 2962, 2927, and 2860 cm^−1^ between BLC–AuNPs, bare AuNPs, and untreated *E. coli*. c) Comparison of integrated area ratios of CH_2_/CH_3_ and CH_2_/CH_2_ peaks observed in BLC‐AuNPs, bare AuNPs, and untreated *E. coli*.

The analysis focused on the asymmetric and symmetric ν(C—H) stretching vibrations of methylene (CH_2_) and methyl (CH_3_) groups in lipid acyl chains of the cell membrane (3030–2800 cm^−1^), which are referred to as ν(CH_2_) and ν(CH_3_), respectively. Variations in peak position and intensity of these ν(C—H) stretching vibrations are known to reflect changes in the lipid ordering and membrane phase state.^[^ ^92^, ^93^, ^94^, ^95^ ^]^ To construct a chemical map, the amide I band at ≈1650 cm^−1^ was first integrated to reveal protein‐rich regions as indicated in red (i.e., high intensity), corresponding to the positions of *E. coli* cells (**Figure** **6**a). Hierarchical cluster analysis (HCA) was subsequently applied to the lipid spectral region (3030–2800 cm^−1^), and average second derivative spectra was extracted from the specific clusters identified as *E. coli* cells. These average second derivative spectra were then inverted and compared across control (untreated) *E. coli*, bare AuNP‐treated, and BLC‐AuNP‐treated cells (Figure 6b).

Interestingly, significant peak shifts were observed only for methylene (CH_2_) stretching modes, whereas those associated with methyl (CH_3_) groups remained unchanged. In particular, the asymmetric ν_as_(CH_2_) stretching mode shifted from 2929 cm^−1^ in control *E. coli* to 2925 cm^−1^ in bare AuNP‐treated cells, while remaining at the same position (2929 cm^−1^) for BLC‐AuNP‐treated cells. Similarly, the symmetric ν_s_(CH_2_) stretching modes shifted from 2862 cm^−1^ (control) to 2864 cm^−1^ (bare AuNPs) and 2860 cm^−1^ (BLC‐AuNPs). Overall, BLC‐AuNP exposure induced a shift towards lower wavenumbers (red shift) in ν_as_(CH_2_) mode compared with the untreated cells, suggesting tighter lipid packing and a more ordered, gel‐like membrane state.^[^ ^94^ ^]^ In contrast, bare AuNPs caused opposing effects, with a red shift of ν_as_(CH_2_) mode, but a blue shift of ν_s_(CH_2_) mode compared with those of the control *E. coli*, indicating complex and potentially destabilizing perturbations to membrane order.

Further evaluation of relative peak area ratios (normalized to the stable ν_as_(CH_3_) peak of the methyl groups) revealed that the BLC‐AuNP treatment markedly increased intensities of both ν_s_(CH_2_) and ν_as_(CH_2_) modes, compared with their corresponding modes observed in the control group (Figure 6c). In contrast, exposure to the bare AuNPs led to substantial intensity reduction of these two modes. A decreased ratio of asymmetric ν_as_(CH_2_) against asymmetric ν_as_(CH_3_), as observed with the bare‐AuNP exposure, has been in some cases associated with the degradation of lipids and the potential loss of CH_2_.^[^ ^95^, ^96^ ^]^ In addition, it was found that the ν_as_(CH_2_)/ν_s_(CH_2_) ratio was lowest for BLC‐AuNPs, and highest for bare AuNPs (Figure 6c). The decrease in ν_as_(CH_2_)/ν_s_(CH_2_) shows further evidence of membrane disruption in samples exposed to bare AuNPs.^[^ ^84^ ^]^ In contrast, the addition of bacterial lipids may suggest that lipids are interacting and depositing, or potentially fusing with the membrane of the cells, leading to more stable structures as evidenced by the observed red shift and the increased ratio of CH_2_ peaks.^[^ ^94^ ^]^ Together, these results suggest that the bare AuNPs disrupt membrane structure, while BLC‐AuNPs promote stabilization and reorganization of membrane lipids into a more ordered configuration. Nonetheless, further work is required to fully elucidate the complex chemical mechanisms underlying nanoparticle‐membrane interactions.

The enhanced adhesion observed for BLC‐AuNP‐treated samples likely arises from the biochemical nature of the bacterial lipid coating. In general, the presence of carboxy and phosphate groups in the membrane of micro‐organisms often imparts a negative surface charge for cells such as *E. coli*.^[^ ^91^ ^]^ Since the shell component of our core‐shell system is made of bacterial lipids with a mixture of mostly PE and PG lipids, a negative surface charge is also expected for BLC‐AuNPs. Despite this repulsive force, strong nanoparticle–cell interactions were observed, suggesting the influence of additional biophysical factors that may be present at the membrane interface during re‐exposure. These may include nanoparticle size, surface charge, lipid composition and packing, as well as membrane curvature.^[^ ^97^, ^98^ ^]^ The lipid packing and surface curvature of BLC‐AuNPs can facilitate the optimal curvature for particles to perturb the bacterial membrane by reducing the threshold for binding energy and potentially inducing fusion between the NP shell and bacterial membranes.^[^ ^98^ ^]^ Furthermore, the initial affinity of the BLC‐AuNP to the cell body may be greatly governed by the electrostatic charge difference between particle and cell bodies as well as the composition of the lipids within the outer shell coating and traces of homotypic components such as membrane phospholipids, lipoproteins, and LPS.^[^ ^94^ ^]^ Other extrinsic factors, such as solvent ionic strength, temperature or pH, may also contribute to the observed interaction between BLC‐AuNPs and *E. coli* cells.

### 2.7 Molecular Dynamics (MD) Simulations

**Figure 7:**
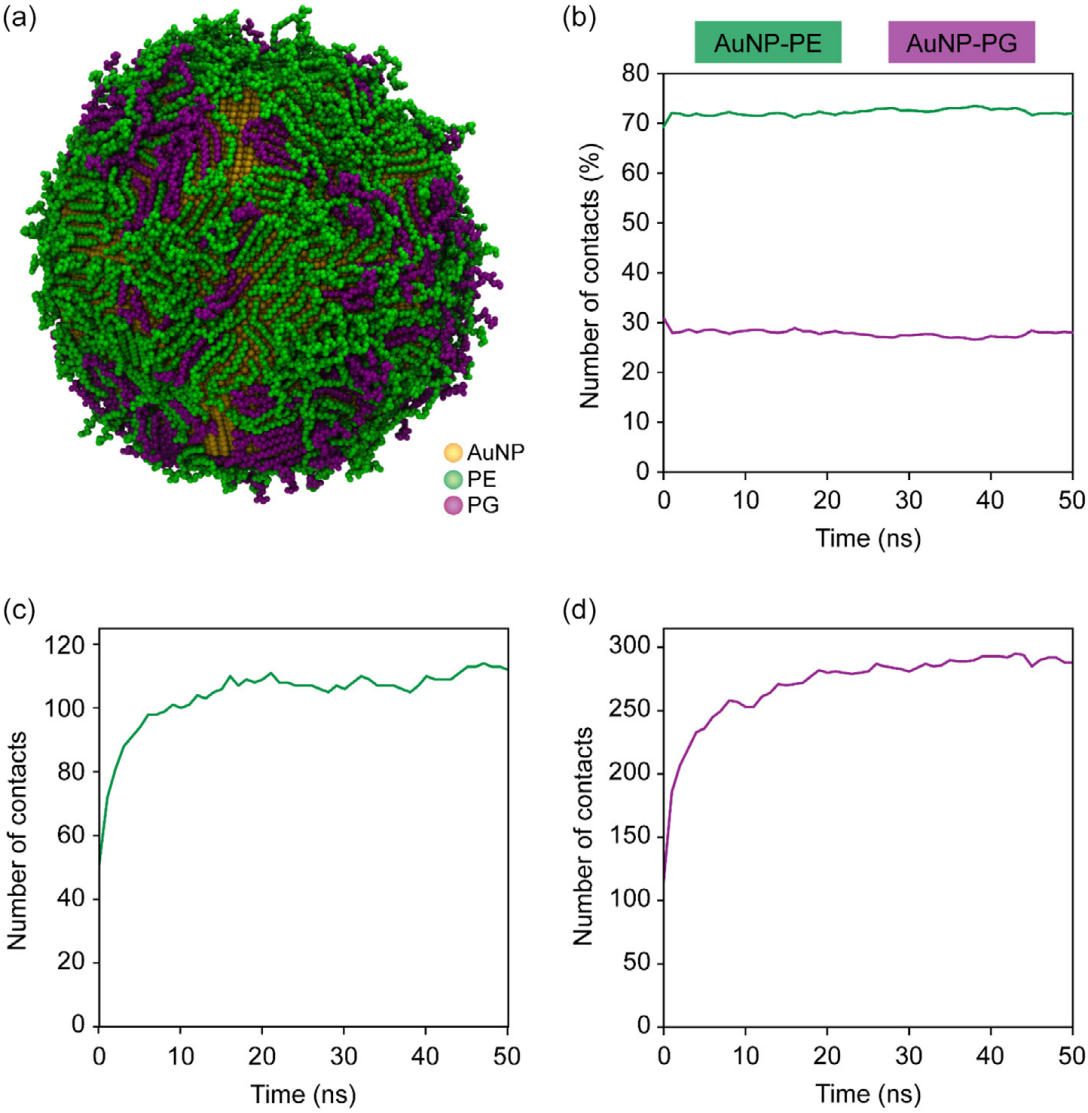
Lipid interaction dynamics in the AuNP–PE–PG system. a) Representative MD simulation snapshot of the AuNP–PE–PG system after 50 ns, showing lipid organization around the AuNPs. b) Percentage of contacts between the AuNPs and each lipid type (PE and PG) over 50 ns simulation. Time evolution of c) AuNP‐PE and d) AuNP‐PG contacts.

MD simulations were conducted to explore the interactions of PE and PG lipids with AuNPs at an atomistic level. It similarly supports the conclusion regarding the formation of a monolayer as the shell component of BLC‐AuNPs. These simulations were specifically designed to replicate the experimental system, facilitating a direct comparison between the computational and experimental results. As such, the simulated systems included solvated AuNPs capped with PE and PG lipids. Adjustments to the pairwise nonbonded interactions between gold and all other atoms were made to reproduce the experimental coverage and exchange mechanisms.^[^ ^99^, ^100^ ^]^ MD simulations of the PE and PG lipids‐capped AuNPs were systematically monitored until the number of lipid–AuNP contacts reached a steady value, which occurred in under 50 ns. This helped develop an understanding of the behavior and interactions of the lipids surrounding AuNPs. Snapshots taken after 50 ns provide a visual representation of the interaction dynamics and the behavior of lipids around the AuNPs (**Figure** **7**a). Quantitative analysis of the AuNP–lipid interactions revealed a consistently higher number of contacts with PE compared with PG over the 50 ns simulation, roughly corresponding to the lipid ratio of 75:25. As shown in Figure 7b, PE contributed ≈72% of the total AuNP–lipid contacts, while PG contributed around 27%, with minimal fluctuation over time. The number of AuNP–PE contacts stabilized around ≈280 (Figure 7c), whereas the number of AuNP–PG contacts plateaued around ≈110 (Figure 7d).

The structure of the lipid corona observed in the simulation is consistent with a monolayer‐type assembly, rather than a bilayer. Furthermore, MD simulations show that most lipid molecules are laid flat onto the surface of AuNPs. This is consistent with prior experimental results obtained from SAXS. The close agreement between simulation and experiment supports the notion that lipid‐coated AuNPs preferentially form monolayers, possibly due to the sharp edges and vertices between facets of the AuNPs and the lack of an appropriate environment to stabilize a bilayer structure. Interestingly, fusion between highly fluid vesicles, such as fusogenic liposomes or OMVs with bacterial cells, typically involves bilayer–bilayer interactions, and very little research has so far been dedicated to monolayer–bilayer fusion mechanisms, which may relate to the interaction observed between our constructed BLC‐AuNPs and *E. coli*.^[^ ^101^, ^102^, ^103^ ^]^ The classic ‘stalk’ structure formation during bilayer–bilayer fusion is energy intensive and requires specific extrinsic conditions to overcome the hydration barrier and form a pore in between the cell membrane and a nominal bilayer, essentially necessitating the reorganization of the inner bilayer leaflet for successful fusion.^[^ ^104^ ^]^ However, for a system involving a monolayer–bilayer, the energy barrier to initiate fusion may prove to be lower.^[^ ^102^ ^]^ More extensive research is needed to understand the underlying energy barriers in monolayer‐bilayer systems.

Often, the Derjaguin, Landau, Verwey, and Overbeek (DLVO) theory has been used to describe the fusion of OMVs, assuming particles and cells as two approaching spherical bodies. DLVO dictates that most forces governing the attraction‐repulsion of two spherical bodies can be simplified to a sum of London van der Waals and electrostatic repulsion potentials.^[^ ^101^, ^105^, ^106^ ^]^ Notably, the involvement of divalent cations such as Ca^2+^ and Mg^2+^ can affect membrane fluctuations, cause steeper polarization, and increased electrostatic strength between membranes. Often, the presence of divalent cations can lead to deviations in DLVO theory; however, some OMV systems were observed to deviate from DLVO despite only containing monovalent cations.^[^ ^6^ ^]^ Similarly to our system, the complexity of the lipid shell on BLC‐AuNPs may involve a myriad of additional factors affecting its electrostatic interactions, and driving its affinity to cells that can be further explored for a more precise understanding of adhesion mechanisms.

## 3 Conclusion

In this work, the enhancement in adhesion and drug delivery capabilities of bacterial lipid‐coated AuNPs was investigated. It was hypothesized that biomimetic cell membrane derived lipid coatings could potentially have a Trojan‐horse‐like effect when combined with inorganic nanoparticles such as AuNPs, in a hybrid core‐shell lipid system. This hypothesis was put to the test by synthesizing BLC‐AuNPs and re‐exposing them to *E. coli* cells to investigate the nature of particle‐cell interactions compared to uncoated AuNPs near the bacterial membrane interface. CLSM and SEM visually confirmed the presence of BLC‐AuNPs within proximity and on the surface of cells, showing a distinct pattern difference in adhesion between samples treated with BLC‐AuNPs and bare AuNPs. Additionally, a significant increase in cell surface coverage in samples treated with BLC‐AuNPs further highlighted the extent of enhancement in particle‐cell adhesion with the presence of bacterial lipid coatings.

Furthermore, chemical changes to the lipid region were analyzed using multivariable HCA of high‐resolution IR spectra. Shifts in the methylene region, as well as large increases in peak area and intensity ratio in the lipid region suggested changes to the lipid structure of the cell membrane, suggesting potential fusion of BLC‐AuNPs with the *E. coli* outer membrane. The nature of the shell coating on BLC‐AuNP was investigated through small angle X‐ray scattering at the Australian Synchrotron. Both SAXS and MD simulated models estimated the formation of a monolayer on BLC‐AuNPs at full coverage lipid concentration, and increasing aggregation in the low‐Q region at higher lipid concentrations of the SAXS plot. Overall, although Van der Waals and electrostatic forces are thought to largely govern the particle‐cell interactions, some intrinsic properties of the membrane derived BLC‐AuNP shell may further enhance the observed adhesion and potential particle‐cell fusion. More extensive research and simulation of the biophysical interaction between cell membrane lipid‐coated particles and their parent cells are required to establish a better understanding of the observed enhancement in particle‐cell adhesion.

## 4 Experimental Section

4.1

4.1.1

##### Materials

LB Agar, LB broth (MILLER) for microbiology GranuCult, phosphate buffer saline (PBS), chloroform (CHCl_3_), sodium chloride (NaCl), gold(III) chloride hydrate (HAuCl_4_.xH_2_O), ammonium acetate (C_2_H_7_NO_2_), and trisodium citrate dihydrate ((CH_2_COONa)_2_ 2H_2_O) were purchased from Sigma‐Aldrich (Australia). The 4′,6‐diamidino‐2‐phenylindole (DAPI), methanol (CH_3_OH), and dichloromethane (DCM) were purchased from Thermo Fisher Scientific (Australia). Rhodamine‐PE 18:1 (Liss Rhod PE 810 150) was purchased from Avanti Research (USA). Formaldehyde/glutaraldehyde fixative (3% in 0.1 M sodium cacodylate buffer with a pH = 7.4) was purchased from Electron Microscopy Sciences (USA).

##### Bacterial Culture

*E. coli* (DH5α) strain was cultured on LB agar and incubated overnight at 37 °C. Bacterial colonies were inoculated into LB broth and further left to grow at 37 °C for 48 h on a rotary shaker (200 rpm). Subsequently, the LB medium containing cells was centrifuged at 5000 × *g* for 5 mins (LabCo centrifuge with 400.004.409 rotor) to pellet the bacterial cells. The resulting pellet was washed three times with 200 μL PBS (pH 7) while being centrifuged between each wash at 5000 g for 5 mins. Bacteria were resuspended in (200 uL) PBS once again prior to lipid extraction.

##### Lipid Extraction from Bacteria

Lipid extraction was performed following a modified Folch method.^[^ ^48^ ^]^ The extraction solution was prepared using chloroform, methanol, and water in a 2:1:1 (v/v/v) ratio, with PBS resuspended bacterial samples serving as the aqueous component. To ensure adequate agitation and transference of lipids from the aqueous to organic phase, stainless steel beads (1.8–3 mm diameter) were added to the extraction mixture prior to being vortexed. This technique represents a minor adaptation of the bead‐beating method described earlier.^[^ ^107^ ^]^ Thereafter, the extraction mixture was vortexed for 20 cycles, each lasting 30 s, to promote thorough mixing. Following agitation, the solution was allowed to separate into respective aqueous (methanol: water) and organic (chloroform) layers. The top aqueous layer containing cell waste was removed carefully, leaving the extracted bacterial lipids in the chloroform layer at the bottom. The organic layer was subsequently washed with 500 μL of a 1:1 v/v methanol‐water mixture for three to five cycles. The lipid containing purified organic layer was collected and dried under running nitrogen (N_2_) gas to form a thin lipid film. Lastly, the thin film was left within a benchtop vacuum desiccator for 2 h to ensure complete dehydration before being stored at −20 °C for future use.

##### Bacterial Liposome Preparation

The thin‐film lipids were initially rehydrated with a 150 mM NaCl buffer to make up bulk solution samples with concentrations of 3.6, 0.66, and 0.5 mg mL^−1^. The mixture was then briefly vortexed, and bath sonicated at 55 °C for 60 min to redisperse the lipids into solution. A freeze‐thaw cycle was performed by immediately placing redispersed lipid solution in a −20 °C freezer for 45 min to chill, followed by another 60 min of bath sonication at 55 °C to form multilamellar vesicles (MUVs). The MUVs containing solution was then manually extruded using Avanti Mini Extruder (Avanti Research, USA). The solution was passed back and forth through a 200 nm polycarbonate filter at least 21 times to ensure the formation of 200 nm single unilamellar vesicles.

Rhodamine‐PE (Rh‐PE, Avanti 18:1 Liss Rhod PE) was incorporated into the thin film of lipids prior to the extrusion of rhodamine containing samples prepared for imaging studies. Rh‐PE was added to the thin film of bacterial lipids at a 1% molar concentration. The two were dissolved and combined in chloroform. The solvent was dried to a thin film under N_2_ gas, placed under vacuum and resuspended/extruded through manual extrusion, following the same methodologies used to prepare non‐rhodamine containing bacterial liposomes.

##### Synthesis of AuNPs

Turkevich method was followed with slight modifications to synthesize AuNPs. The reduction of Au was initiated by combining pre‐heated (70 °C) solutions of trisodium citrate and gold salt dissolved in MilliQ water at a 1:7.87 molar ratio. In this case, 26.4 mg of gold precursor and 157.9 mg of sodium citrate were separately dissolved in 35 mL of MilliQ water and preheated to 70 °C prior to being combined. The size of AuNP was dependent on the temperature and kinetics of the reaction.^[^ ^72^ ^]^ As such, the combined solution was placed on a hotplate and kept at a constant 80 °C temperature for 60 min to synthesize 10–15 nm AuNP dispersion. The color change of the reaction mixture was closely monitored throughout the process, to ensure that it reflects the size range associated with LSPR.^[^ ^77^ ^]^

##### Synthesis of Bacterial Lipid‐Coated AuNPs (BLC‐AuNPs)

BLC‐AuNPs were made from combining 20 or 7 μL of bulk lipid with 400 μL or 1 mL of AuNPs, respectively, to make a final concentration of 0.029 ± 0.0036 mg mL^−1^ lipid BLC‐AuNP solution for particle‐cell experiments (Table S2 and Table S6, Supporting Information). The mixture was then sonicated in a water bath at 55 °C for 45 min to induce vesicle fusion resulting in the formation of BLC‐AuNPs. Given the heterogenous composition of lipids in the *E. coli* lipid extracts, a nominal temperature (55 °C) above the literature‐reported *E. coli* membrane Tm (≈30 ± 3 °C) was chosen.^[^ ^108^ ^]^ This Tm value serves to induce a collective structural re‐organization of the lipid mixture within the *E. coli* extract, rather than inducing a phase transition of select lipid species. The selected temperature could ensure sufficient kinetic energy for vesicle fusion while being cautious of potential PE bilayer‐to‐hexagonal phase transition at ≈60–65 °C.^[^ ^109^ ^]^ A series of calculations were completed to estimate the molar concentration of lipid solution required to sufficiently coat the surface area of AuNPs in solution prior to sonication (Table S2, Supporting Information). The lipids were added in excess of the calculated minimum (5.2 ± 1.5‐fold) to ensure adequate coverage of all AuNPs (Table S6, Supporting Information). It also helped to limit potential aggregation, which can be seen by changes in the color of the colloidal solution. Finally, BLC‐AuNPs were allowed to cool at room temperature for at least 30 mins before experimentation.

##### Characterization

UV‐Vis spectroscopy. The NP were scanned using a UV‐Vis spectrophotometer. Both AuNP and BLC‐AuNP solutions (3 mL) were transferred to standard 10 mm glass cuvette to capture wavelength absorbances between 300 and 800 nm using the CARY 3500 UV‐Vis spectrophotometer (Agilent Technologies, Germany). Here, MilliQ water was used as reference.

DLS. The hydrodynamic diameter of AuNPs, BLC‐AuNPs, and extruded liposomes were measured by DLS using Zetasizer Nano ZS (Malvern, UK) fitted with a 633 nm laser.

FTIR. Bacterial lipids which were previously dehydrated into a thin film were transferred directly to the sensor on the Burker Alpha II FTIR (USA) to generate a spectrum ranging between 4000 and 500 cm^−1^. Spectrum was normalized and smoothed 5 times using the Savitsky‐Golay method.

MALDI‐TOF. MALDI‐TOF was performed on bacterial lipids in positive ion mode [M + H]^+^. A sample of thin film lipids was dissolved in methanol (5.7 mg mL^−1^) and drop casted onto ground steel target plates and scanned using the Burker Autoflex Speed MALDI‐TOF mass spectrometer (Bruker Daltonik, Bremen, Germany). A 2,5‐dihydroxybenzoic acid **(**DHB) matrix (Bruker Daltonik, Bremen, Germany) was used initially with the sample being drop casted on top upon matrix dehydration in a 1:1 ratio. Spectrum was taken between 300 and 1500 m/z.

LC‐MS. Lipid characterization was accomplished using 1260 Infinity II high performance liquid chromatography (LC) instrument (Agilent Technologies, Germany). The system consisted of a quaternary pump G7111B, multi‐sampler G7167A, multicolumn thermostat G7116A and a G7117C diode array detector. Analytes were separated using the Agilent Eclipse XDB‐C18 column (dimensions: 4.6 mm ID 250 × mm, with a pore size of 80 Å and Zorbax Rx‐SIL silica). Ionization was set to negative ESI [M‐H]^−^ mode. The thin film lipid extract sample was dissolved in methanol for qualitative analysis of phospholipids. The mobile phase consisted of the gradient elution of 20 mM ammonium acetate (solvent A), DCM (solvent B), and methanol (solvent C) to various mixture ratios within a 35 min interval. The injection volume of the sample was 10 μL. The LC‐MS acquisition was initiated with a 1:1:4.67 ratio of solvent A, B, and C (15, 15 and 70% respectively) and a flow rate of 0.5 mL min^−1^. Between 2 and 30 min, the elution of solvent B was gradually increased to a maximum of 70%, with a gradual decrease of solvents A and C to 15% respectively. Solvents were kept constant between 30 and 31 min following a graduate change to a final mixture of 15% solvent A, 25% solvent B, and 60% solvent C by the 35 min mark.

##### Confocal Scanning Laser Microscopy (CLSM)

Confocal microscopy was performed using the ZEISS LSM 880 Airyscan upright microscope. For bacterial sample preparation, *E. coli* was suspended in PBS at an optical density (OD) of 0.2. The cells were then stained with DAPI (300 nM), a blue‐fluorescent DNA stain, before commencing imaging. To prepare fluorescing BLC‐AuNPs, Rh‐PE was incorporated into the lipid thin film at a 1% molar percentage of the dried lipids in accordance with previous methods of fluorescent lipid staining. To do this, Rh‐PE dissolved in chloroform was mixed with previously prepared bacterial thin‐film lipids and dried under N_2_ gas and subsequently rehydrated and extruded in accordance with the standard method of liposome preparation as described previously. Lipid fluorescent BLC‐AuNPs were synthesized through the previously discussed vesicle fusion method. Bare AuNPs were directly exposed to cells at the same ratio and conditions as BLC‐AuNPs. The auto‐fluorescing property of AuNPs allows for detection of aggregates without the need for staining.

During CLSM imaging, the water immersion lens was submerged in *E. coli* suspended in PBS. While imaging, both 405 and 561 nm lasers were enabled as Rh‐PE is expected to reach maximum emission of 580 nm (red) with DAPI reaching a max emission of 461 nm (blue). Control images of cells were taken prior to particle‐cell exposure. Prepared particles (bare AuNPs and BLC‐AuNPs) were slowly and gently injected into the confocal disk containing *E. coli* (0.2 OD) at a v/v ratio of 1:10. The cell focal point was reconfigured, and the position of the cells was ensured to match the control cell images prior to imaging. Snapshots were taken immediately after particle exposure, and every 30 min thereafter for a total of 120 min. The red channel intensity for control AuNP‐cell exposure was artificially increased to acquire the very last snapshot (at 120 min) to ensure the absence of false negative results.

##### Scanning Electron Microscopy (SEM)

To visualize particle‐cell interactions, SEM was performed on cell samples exposed to either bare control AuNPs or BLC‐AuNPs. Sample preparation for the SEM closely followed that of the CLSM experiments without the use of fluorescent dyes and with a 1:1 v/v ratio of particle to cell solution volume. 2 h after particle‐cell incubation, the mixed solution was fixed using 3% formaldehyde/glutaraldehyde fixative in 0.1 M sodium cacodylate buffer with a pH 7.4 (Electron Microscopy Sciences) at a 1:1 ratio. The sample was left in the fixative for 1 h followed by ethanol wash. The ethanol washing was done in a series of increasing concentrations: 30%, 50%, 80%, 90%, and 100% to wash the fixed solution on silicon wafers. Samples were left overnight and dried under N_2_ gas the following day for imaging. The FEI Verios 460 L XHR‐SEM scanning electron microscope was set to a low voltage (1–3 kV) and a 3.1–6.3 pA current in immersion mode for cell imaging.

##### Synchrotron Macro‐ATR‐FTIR Microspectroscopy

Synchrotron Fourier transform infrared (s‐FTIR) measurements were performed on the Infrared Microspectroscopy (IRM) beamline at the Australian Synchrotron, using a Bruker Vertex 80v spectrometer coupled to a Hyperion 3000 FTIR microscope and a liquid nitrogen‐cooled narrow‐band mercury cadmium telluride (MCT) detector (Bruker Optik GmbH, Ettlingen, Germany). All the s‐FTIR spectra were collected over the range of 3900–750 at 4 cm^−1^ spectral resolution. Default acquisition parameters included Blackman‐Harris 3‐term apodization, Mertz phase correction, and a zero‐filling factor of 2, operated via OPUS 8.0 software suite (Bruker Optik GmbH, Ettlingen, Germany).

Specifically, the microbial membrane lipid composition following 2 h exposure of *E. coli* cells to BLC‐AuNPs was analyzed and imaged in macro ATR‐FTIR imaging mode, using an in‐house developed macro ATR‐FTIR device that was equipped with a 250 μm‐diameter facet germanium (Ge) ATR crystal (*n*_Ge_ = 4.0) and a 20× IR objective (NA = 0.60; Bruker Optik GmbH, Ettlingen, Germany).^[^ ^91^ ^]^ The unique combination of the high refractive index property of the Ge ATR crystal and the high numerical aperture (NA) objective used in this device, when coupled to the synchrotron‐IR beam, enabled high‐resolution chemical imaging of the particle‐cell interface with a minimal pixel resolution of 250 nm.

Microbial samples and NP treatments (BLC‐ and bare AuNPs) were prepared as described in the SEM experiments. Nanoparticles were incubated with *E. coli* cells in PBS (OD = 0.2) on silicon wafer substrates for 2 h, followed by drying under N_2_ gas. Samples were washed three times with Milli‐Q water to remove excess salts and cellular debris, prior to mounting onto an aluminum disc holder. A background spectrum was collected in air, using 4 cm^−^^1^ spectral resolution and 256 co‐added scans. Subsequent mapping measurement was conducted using a projected aperture of 3.13 μm in diameter per pixel at a step interval of 0.5 μm, and eight co‐added scans.

The acquired spectral maps were pre‐processed using atmospheric compensation in OPUS 8.0 (Bruker). Noise reduction, second derivatization, and vector normalization were then applied before performing hierarchical cluster analysis (HCA) in the two spectral ranges (i.e. 3030–2800 and 1800–1000 cm^−1^) using CytoSpec v. 1.4.02 (Cytospec Inc., Boston, MA, USA), to capture changes in the key biochemical features of the *E. coli* cells.

##### Synchrotron Small Angle X‐Ray Scattering (SAXS) Measurements

The SAXS components of this work were performed on the BioSAXS beamline at the Australian Synchrotron, part of ANSTO. A Pilatus3X‐2M detector (Dectris ltd) was used to measure the small angle scattering profiles of an AuNP solution undergoing sequential additions of BLC lipid dispersion. The instrument was operated with an X‐ray wavelength of 1.00 Å (photon energy = 12.4 keV) and a sample to detector distance was 2865 mm. This afforded a measured Q‐range between 7.1 × 10^−3^ and 5.0 × 10^−1^ Å^−1^. A 1.0 mm diameter capillary was held in the X‐ray beam path, and a citrate‐capped AuNP solution ([AuNPs] = 0.11 mg mL^−1^) in a vial was circulated through the capillary and back into the vial using a peristaltic pump. A fraction of a 5 mL solution of bare‐AuNPs was continually flowed through the capillary, while the remainder was stirred in the vial. Bacterial liposomes were progressively added to the bare‐AuNP suspension to make final concentrations of 1.33 × 10^−3^, 2.65 × 10^−3^, 5.30 × 10^−3^, 1.06 × 10^−2^, 2.09 × 10^−2^, and 3.12 × 10^−2^ mg mL^−1^ BLC‐AuNPs. After the addition of each aliquot of BLC lipid dispersion, 1 s acquisitions were taken to monitor the changes in NP structure after each injection. Once the samples had equilibrated, another lipid injection was performed. Data processing including normalization of data on an absolute scale and integration of two‐dimensional SAXS patterns, was performed by Fast Azimuthal Integration using Python (PyFAI) with custom scripts written for the BioSAXS beamline. Further fitting and analysis of the scattering data were conducted with SasView 6.0.0 and 6.1.0 software.^[^ ^110^ ^,^ ^111^ ^]^ A background measurement of 150 mM NaCl solution in the measurement capillary was subtracted from the sample data prior to modelling. Shape independent fractal, and core‐shell fractal models along with Sphere and core‐shell sphere models were fitted to the reduced and background subtracted data to obtain an optimal model representing the BLC‐AuNP system.

##### Molecular Dynamics (MD) Simulations

The 13 nm AuNPs were designed using the Wulff Construction method in the CHARMM‐GUI Nanomaterial Modeller, specifying a 6.5 nm radius and incorporating Miller indices of 100, 110, and 111.^[^ ^113^, ^114^, ^115^ ^]^ A single lipid structure of the PE and PG lipids was created using the CHARMM‐GUI Membrane Builder.^[^ ^115^ ^]^ Subsequently, 600 PE and 200 PG lipids to reflect a 75:25 ratio were arranged around the 13 nm AuNPs using the PACKMOL software.^[^ ^7^ ^]^

Each system was equilibrated within a cubic simulation box (31.2 × 31.2 × 31.2 nm^3^) containing one AuNP, 800 lipid molecules, and CHARMM TIP3P water, with 0.15 M NaCl and neutralizing counterions for 125 ps. MD simulations were conducted until the number of lipid – AuNP contacts reached a steady value, which occurred in under 50 ns, using the MD code GROMACS 2023.^[^ ^116^ ^]^ The modified INTERFACE forcefield for AuNPs with the CHARMM forcefield for lipids were applied to all atoms.^[^ ^100^, ^117^, ^118^, ^119^ ^]^

Short‐range nonbonded interactions were handled using the Verlet cutoff scheme, with a cutoff distance of 12 Å and a switch distance of 10 Å. Long‐range Coulomb interactions beyond 12 Å were computed using the particle mesh Ewald (PME) method. The system was maintained at a reference temperature of 298.15 K during equilibration using the Berendsen temperature coupling algorithm. The production simulations were conducted at the same temperature, controlled using the Nosé−Hoover weak coupling dynamics, to establish an NPT ensemble, where the number of particles (N), pressure (P), and temperature (T) were kept constant. Constant pressure was maintained using the Parrinello–Rahman barostat. A time step of 2 fs was used with atomic coordinates saved every 10 ps. All the other simulation parameters followed the default settings provided by CHARMM‐GUI.^[^ ^99^ ^]^ Analysis was conducted using the software Visual Molecular Dynamics (VMD).^[^ ^120^ ^]^

##### Statistical Analysis

DLS size was reported as intensity‐weighted distributions (Z‐average) (*n* = 5). Both Z‐average and PDI values were reported as mean ± SD. TEM particle size distribution was (*n* = 364) were presented as mean ± SD, with normal distribution calculated and plotted using the Origin Pro software (Figure S2, Supporting Information). MALDI‐TOF data were reported as a percentage of the total absorption across the acquired spectrum. Prior to plotting, MALDI‐TOF data were matrix‐subtracted. For LC‐MS, data from each peak of the TIC was sorted by their relative abundance, and the top 10% strongest readings for each peak were combined and plotted as a percentage of the absorbance sum. Plotted LC‐MS data consisted of relative intensities above a 0.1% threshold (Figure S1, Supporting Information). Lipid concentrations and lipid coating factors were reported as mean ± SD (Table S2, Supporting Information). To investigate the statistical significance of the cell surface coverage percentages found via SEM imaging, a Mann‐Whitney *U*‐test with T‐tie correction was implemented. *E. coli* samples treated with either BLC‐AuNPs or AuNPs were compared. Each group contained a sample size of 13 (*n*_BLC‐AuNPs_ = 13, *n*_AuNPs_ = 13) (Table S7–S9, Supporting Information). Differences were considered statistically significant when the probability value (*P* value) was less than 0.05. Datasets were sorted from lowest to highest value prior to finding the median.

## Supporting Information

Supporting Information is available from the Wiley Online Library or from the author.

## Conflict of Interest

The authors declare no conflict of interest.

## Supporting information

Supplementary Material

## Data Availability

The data that support the findings of this study are available in the supplementary material of this article.
