# Supplementary material for: Harnessing Bacterial Lipid Coatings on Gold Nanoparticles for Enhanced Cell Adhesion Applications
Source: Small Sci. 2026 Feb 17;6(2):e202500584. doi: 10.1002/smsc.202500584 (PMC12911120; doi:10.1002/smsc.202500584)
Supplement: Supplementary file 1 — Supplementary Material [file SMSC-6-e202500584-s001.pdf]

Supporting Information

Harnessing Bacterial Lipid Coatings on Gold Nanoparticles for Enhanced Cell Adhesion Applications

Soroosh Gharehgozlo,[1] Shakil Ahmed Polash,[1] Kalpani A. Mirihana,[1] Jakub Matusiak,[2] Lauren Giles,[1] Pierre Vaillant,[1] Shreehari Kodakkat,[1] Serena Ch'ng,[1] Rowan Penman,[1] Samuel Cheeseman,[3,4] Lydon Alexandrou,[1] Jitraporn Vongsvivut,[5] Andrew J. Clulow,[5] Andrew J. Christofferson,[1] Paul A. Ramsland,[1,6,7] Saffron J. Bryant,[1*] and Aaron Elbourne[1*]*

[1]School of Science, STEM College, RMIT University, Melbourne Victoria 3001, Australia
[2]Department of Construction Materials Engineering and Geoengineering, Faculty of Civil Engineering and Architecture, Lublin University of Technology, Nadbystrzycka 40, 20-618, Lublin, Poland
[3]Department of Biomedical Engineering, Faculty of Engineering and Information Technology, The University of Melbourne, Parkville, Victoria, 3010, Australia
[4]The Graeme Clark Institute, The University of Melbourne, Parkville, Victoria, 3010 Australia
[5]Australian Synchrotron, ANSTO, 800 Blackburn Road, Clayton, Victoria 3168, Australia
[6]Department of Immunology, Monash University, Melbourne, VIC 3004, Australia
[7]Department of Surgery, Austin Health, University of Melbourne, Heidelberg, VIC 3084, Australia

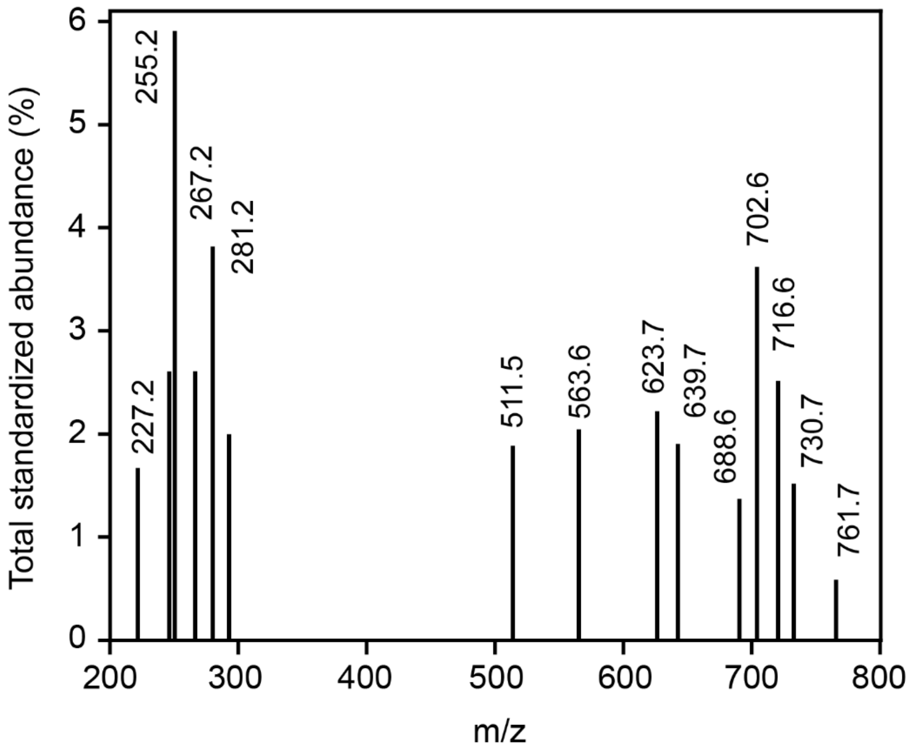

Figure S1. LC-MS of top 10% most abundant relative intensity readings.

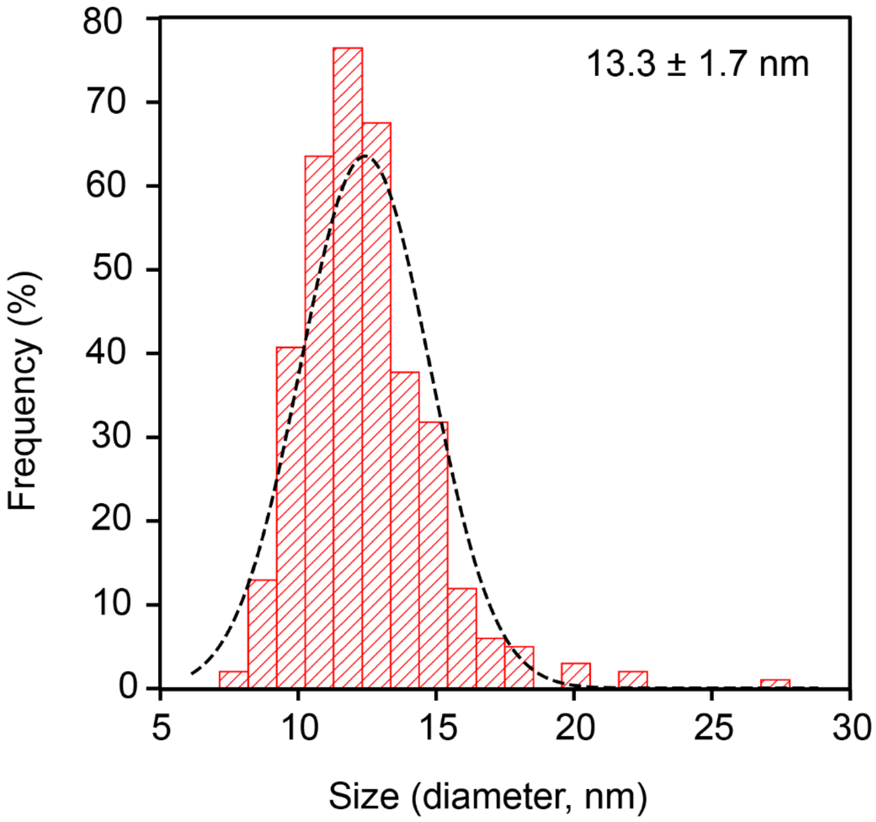

Figure S2. Particle size distribution of Cit-AuNPs. (n = 364)

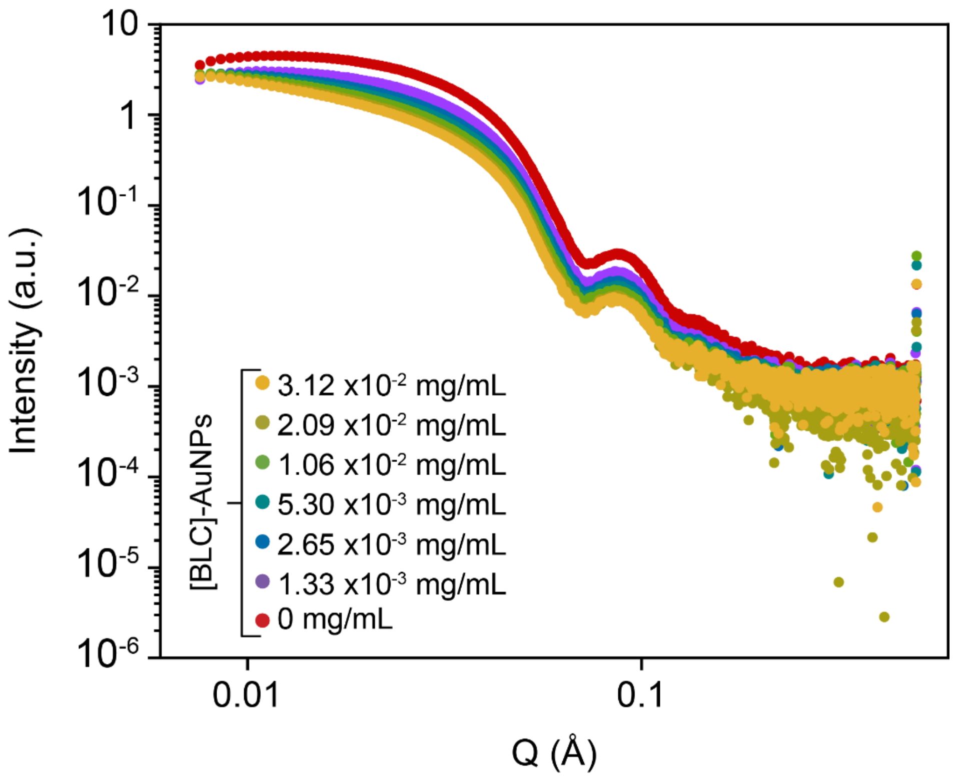

Figure S3. Background subtracted SAXS data without intensity offset. Due to some observed particle settling, the reduction in volume fraction can be seen with increasing lipid concentrations. Therefore, volume fraction was left unconstrained during model fitting.

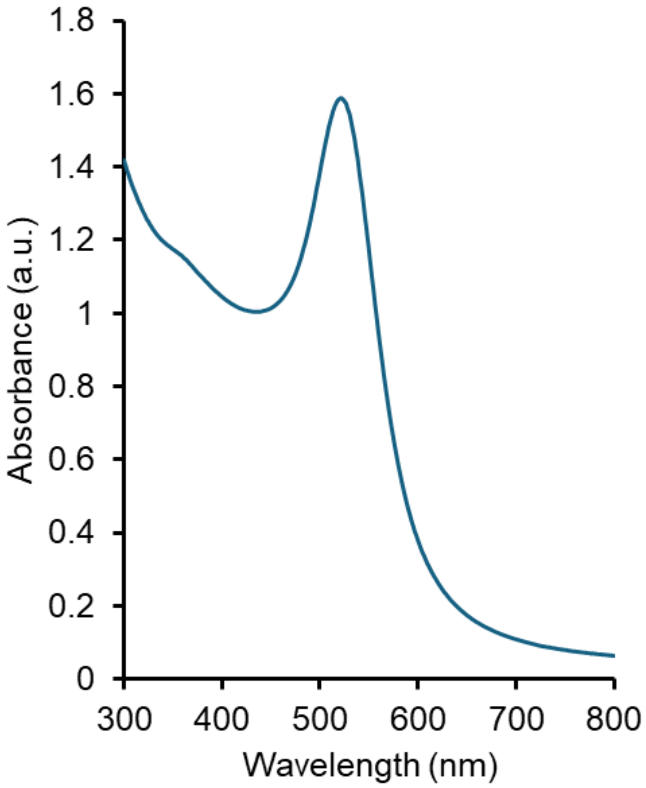

Figure S4. UV-vis spectrum of BLC-AuNPs.

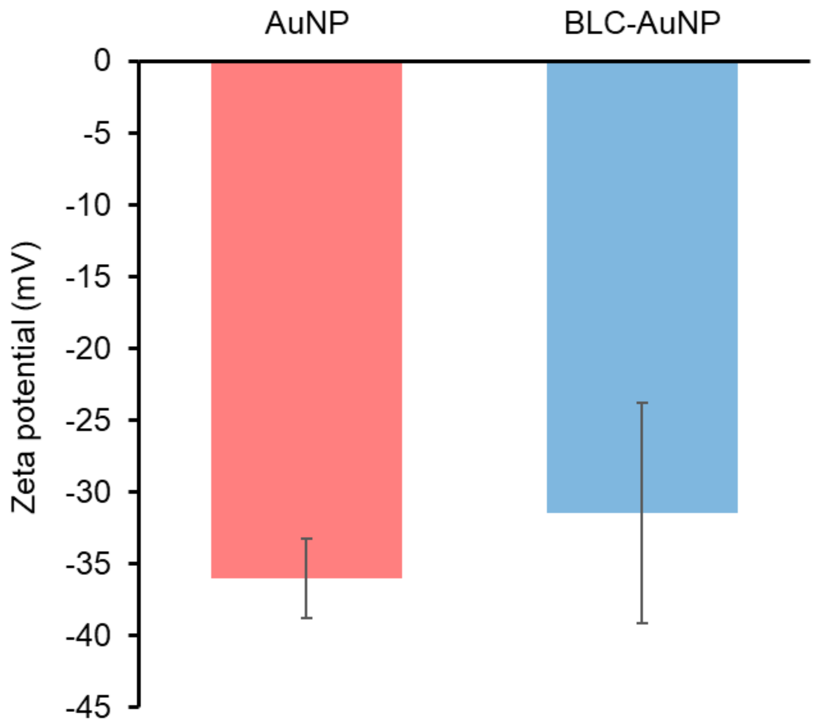

Figure S5. Zeta potential of bare AuNPs and lipid-coated derivative (BLC-AuNPs).

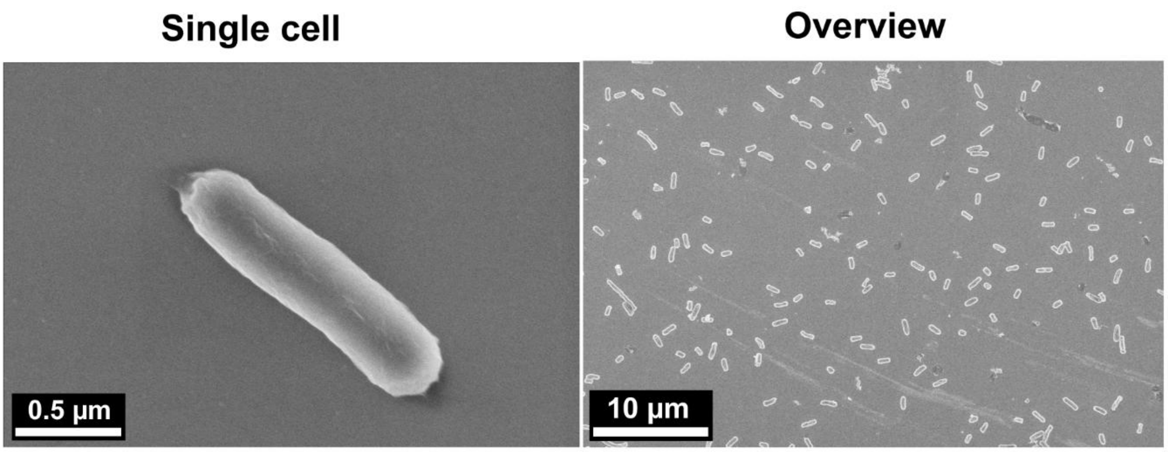

Figure S6. SEM Control *E. coli*

Table S1. Interplanar spacings measured from the SAED pattern of synthesized bare AuNPs.

**Ring number**	**D/2 Measurement**	**1/r (nm)**	**r (nm)**	**r (Å)**	***h k l***
1	8.604	4.302	0.2325	2.32	(111)
2	9.861	4.931	0.2028	2.03	(002)
3	13.897	6.949	0.1439	1.44	(022)
4	16.188	8.094	0.1235	1.24	(113)
5	16.844	8.422	0.1187	1.19	(222)

Table S2. Lipid usage and standardization parameters for BLC- AuNPs characterization across different instruments.

Instrument	Volume of bulk lipids used (mL)	Mass of lipid in solution (mg)	Concentration of lipid bulk used (mg/mL)	AuNPs volume (mL)	Standardized lipid concentration in BLC-AuNPs (mg/mL)	Lipid coating factor (n-Fold)
DLS	0.00699	0.025164	3.6	1	0.024989325	4.034808389
Confocal	0.02	0.01	0.5	0.4	0.023809524	3.844316238
SEM	0.02	0.0132	0.66	0.4	0.031428571	7.903178874
IRM	0.02	0.0132	0.66	0.4	0.031428571	5.074497434
TEM	0.02	0.0132	0.66	0.4	0.031428571	5.074497434
Zeta-Potential	0.05	0.033	0.66	1	0.031428571	5.074497434

Table S3. Thickness parameters from core-shell fractal model.

AuNPs volume (ml)	Concentration (mg/mL)	Shell Thickness (Å)
5	0.00E+00	0
5.005	1.33E-03	0
5.01	2.65E-03	0
5.02	5.30E-03	11.80
5.04	1.06E-02	16.2
5.08	2.09E-02	12
5.12	3.12E-02	9.80

Table S4. Sphere model parameters.

Lipid concentration (mg/mL)	0	0.00133	0.00265
Chi2	54.534	37.396	71.843
scale	3.86E-06	2.39E-06	1.82E-06
scale_err	7.64E-10	5.76E-10	5.30E-10
background	8.00E-04	8.00E-04	8.00E-04
sld	118	118	118
sld_solvent	9.45	9.45	9.45
radius	62.153	62.153	62.153
radius_err	0.006187	-	-
radius.width	0.12	0.12	0.12
radius.nsigmas	1	1	1
radius.npts	0	0	0
Polydispersity	0.12	0.12	0.12

Table S5. Core-shell fractal model parameters

Lipid concentration (mg/mL)	0.0053	0.0106	0.0209	0.0312
Chi2	9.0824	8.2033	7.9753	8.6663
scale	1	1	1	1
background	0.0008	0.0008	0.0008	0.0008
radius	62.153	62.153	62.153	62.153
thickness	11.773	16.183	11.962	9.8105
thickness (Err)	1.5728	1.3467	1.1846	1.1019
sld_core	118	118	118	118
sld_shell	8	8	8	8
sld_shell (Err)	0.43625	0.30285	0.43127	0.54408
sld_solvent	9.45	9.45	9.45	9.45
volfraction	2.03E-06	2.09E-06	1.45E-06	1.19E-06
volfraction (Err)	1.37E-07	1.14E-07	7.41E-08	5.85E-08
fractal_dim	1.5	1.5	1.5	1.5
cor_length	57.902	77.875	92.932	102.31
cor_length (Err)	0.74781	0.9858	1.1885	1.3273
Polydispersity	0.12	0.12	0.12	0.12

Table S6. Mean lipid concentration and lipid coating factor used across experiments

Mean Concentration	0.0291
Standard Deviation	0.0036
Lipid Concentration (mg/mL)	0.02909 ± 0.00365
Lipid coating Factor	
Mean	5.167632634
Standard Deviation	1.452149633
Lipid Coating Factor	5.16763 ± 1.45215

To calculate the minimum concentration for full lipid coverage, the total number and surface area of AuNPs per unit volume is estimated. The volume of the bulk lipid needed to cover the surface area of all AuNPs per unit volume with lipid headgroups is estimated at 6.19×10^{-3} mg/mL. The lipid coating factor for each experiment therefore represents an n-fold increase from the minimum lipid concentration needed to achieve full AuNPs coverage. Notably, due to the complexity of membrane lipids and lipid mass variations in each batch of lipid extracts, these estimates were only used as a guide to approximate the mixing ratio of AuNPs with extracted lipids.

SEM surface coverage

The Mann-Whitney U test was conducted as opposed to the two-factor t-test to compare the BLC-AuNPs and Bare AuNPs populations due to the distribution of data points. Since the surface coverage data was not normally distributed and was under $n<30$, the Mann-Whitney U test provided an ideal evaluation of significance between populations.

Table S7. Bacterial surface coverage percentage (Bare AuNPs)

**Bare AuNPs** – *E. coli* exposure		
Total Cell (pixels)	Covered area on cell (pixels)	% Coverage
43847	12329	28.11823
35227	359	1.019105
36855	0	0
26013	0	0
40127	0	0
33885	0	0
22285	0	0
23899	6480	27.11411
26480	7383	27.88142
34402	501	1.456311
35636	5326	14.94556
29373	0	0
209051	922	0.441041

Table S8- bacterial surface coverage (BLC- AuNPs)

**BLC-AuNPs** – *E. coli* exposure		
Total Cell (pixels)	Covered area on cell (pixels)	% Coverage

11548	10001	86.60374
10630	4741	44.60019
14121	0	0
20125	0	0
21916	13724	62.62092
12677	7215	56.9141
12726	915	7.190005
20628	7744	37.54121
21745	9747	44.8241
12348	9087	73.59086
15388	0	0
10859	0	0
137645	80301	58.33921

Table S9. Mann-Whiteny U test parameters calculated

T1	135
T2	216
U1	125
U2	44
U-Value	44
T-tie correction	82.5
Error tie corrected	18.94201679
Expected U	84.5
Standard error	19.5
Z-value	-2.138103902

Two tailed **p-value** equates to 0.032517.
